# A Bayesian Optimal Adaptive Clinical Trial Design for Sequentially Integrated Therapies

**DOI:** 10.1002/sim.70684

**Published:** 2026-07-30

**Authors:** Yining Li, Jiaying Guo, Samer Gawrieh, Wei Shen, Yong Zang, Wanzhu Tu

**Affiliations:** ^1^ Department of Biostatistics and Health Data Science Indiana University School of Medicine Indianapolis IN USA; ^2^ Eli Lilly and Company Indianapolis IN USA; ^3^ Department of Medicine, Division of Hepatology and Gastroenterology Indiana University School of Medicine Indianapolis IN USA; ^4^ Center for Computational Biology and Bioinformatics Indiana University School of Medicine Indianapolis IN USA

**Keywords:** alcohol‐associated hepatitis, Bayesian adaptive design, clinical trial, integrated therapy, treatment optimization

## Abstract

Clinical syndromes or diseases with complex etiology often require multiple treatments, each addressing one specific aspect of the disease. Such treatments are typically administered sequentially in an integrated fashion. For example, in alcohol‐associated hepatitis (AH), an acute liver disease caused by excessive alcohol drinking, successful treatment requires therapies that reduce liver inflammation during the acute phase, followed by interventions that address the underlying alcohol use disorder (AUD) to achieve optimal outcomes. Selecting an optimal treatment combination, however, presents great challenges in study design due to the involvement of multiple drug combinations and treatment phases. In this paper, we describe a new trial design based on a Bayesian model that connects the outcomes from the acute and subsequent treatment phases. We refer to this design as the Bayesian Optimal Adaptive Design for Sequentially Integrated Therapies (BIT) design. BIT incorporates multiple interim analyses with adaptive stopping rules for both futility and superiority, enhancing efficiency while controlling the family‐wise type I error rate and maximizing statistical power. We illustrate the use of BIT design in a clinical trial comparing the efficacy of integrated therapy for severe AH, with sample size determination and design parameter optimization. Simulation studies show that the design possesses excellent operating characteristics, including experiment‐wise type I error rate control. Although the design's development is motivated by studies of AH treatment, the design framework is broadly applicable to other complex diseases requiring sequentially delivered therapies. R codes are provided for BIT implementation.

## 1 Introduction

In clinical practice, treating complex syndromes often requires multiple therapeutic agents to address distinct clinical needs, delivered in a sequence dictated by the urgency and priority of the underlying conditions. We refer to such treatment strategies as *sequentially integrated therapies*: coordinated treatment programs in which two or more therapeutic components, each targeting a clinically distinct aspect of a single complex underlying disease, are delivered in a biologically mandated sequence across temporally distinct phases of care, with the primary clinical estimand defined over the entire sequence. Specifically, we consider three design features: (i) the components address conceptually different clinical needs, with different mechanisms, different targets, and different time horizons, rather than being sequential lines of the same therapeutic class; (ii) the ordering is determined by disease biology rather than by patient response or investigator preference; and (iii) the relevant outcome is a joint function of all components, so no single component evaluated in isolation captures the therapeutic effect of interest.

A motivating example arises in alcohol‐associated hepatitis (AH), a condition resulting from prolonged and excessive alcohol consumption. Effective management of AH requires addressing two interrelated clinical problems: acute inflammatory liver injury, with substantial short‐term mortality (30‐day mortality 10%–24%; 90‐day mortality up to 44%; Mathurin et al. [1], Thursz et al. [2], Szabo et al. [3]), and the underlying alcohol use disorder (AUD) that precipitated the liver damage. Long‐term survival depends on the effectiveness of both acute‐ and post‐acute therapies, delivered in that order; without effective control of alcohol consumption, survivors of acute AH frequently relapse, leading to recurrent episodes of AH and markedly increased risks of liver decompensation, cirrhosis, and death [4, 5]. The relevant clinical estimand is the overall survival probability under the entire AH‐AUD sequence, not the effect of any single component. A recent expert panel consensus statement [6] has explicitly identified the development of clinical trial designs for such integrated AH‐AUD interventions as a research priority. Section 2 describes this clinical setting in detail.

The design framework is not specifically developed for AH‐AUD treatment trials. Analogous treatment paradigms arise in stage III non‐small‐cell lung cancer, where definitive chemoradiotherapy is followed by maintenance immunotherapy [7]; in acute heart failure, where in‐hospital stabilization is followed by outpatient cardiac rehabilitation [8]; and in sexually transmitted infections, where antimicrobial treatment is followed by partner notification and behavioral risk‐reduction interventions [9]. In each case the components are biologically ordered, address distinct clinical needs, and yield a meaningful outcome only when considered jointly.

Despite the prevalence of such treatment practice, trial designs for evaluating sequentially integrated therapies remain limited, and existing multi‐stage designs do not fully address its specific structure. Multi‐arm multi‐stage (MAMS) trials [10] discontinue ineffective arms at prespecified interim analyses, but each arm is a static treatment without internal phase‐level structure; the “stages” refer to interim analyses for trial‐level decisions rather than to therapeutic phases within a patient's care. A 2×2 factorial design over (acute treatment, post‐acute treatment) would in principle compare integrated combinations, but factorial inference assumes that all components are delivered to all patients, an assumption violated when later‐phase treatment is conditional on earlier‐phase survival. Sequential multiple assignment randomized trials (SMART) and the broader dynamic treatment regime literature [11, 12, 13, 14] target a fundamentally different estimand: optimal *decision rules* that select second‐stage treatment as a function of intermediate response. Two‐stage randomization, which is the identifying feature of SMART, is unnecessary when treatment ordering is biologically mandated, and it can induce collider bias on Phase 1 survival when survivor populations differ across Phase 1 arms. The Bayesian Optimal Phase II framework [15, 16] provides a flexible posterior‐probability‐based decision procedure for single‐endpoint Phase II designs but does not address the conditional survival structure across treatment phases. None of these frameworks directly accommodates the joint estimand defined over an integrated sequence with phase‐dependent conditioning on survival.

Bayesian adaptive designs are well suited to this setting because of their ability to incorporate prior information, accommodate interim decision‐making, and flexibly model complex outcome structures. A substantial literature has developed Bayesian methodology for comparative clinical trials, including posterior probability‐based two‐stage designs [17], practical guidance on decision boundaries [18], Bayesian interim monitoring procedures [19], seamless phase II/III designs [20], predictive probability‐based decision rules [21], Bayesian methods for optimizing treatment regimes [22], and hybrid and generalized designs spanning early to late phases of development [23, 24]. Comprehensive reviews are provided in Thall [25, 26], Lee and Chu [27], Zang and Lee [28]. These methods provide important building blocks but have been motivated primarily by early‐phase oncology trials and do not explicitly address the challenges posed by sequentially integrated therapies, in which distinct agents are delivered across different disease phases within the same patient with later‐phase outcomes contingent on earlier‐phase survival.

In this paper, we propose a Bayesian adaptive design for sequentially integrated therapies, which we refer to as the BIT design. For clarity of exposition we present the methodology in the context of an AH‐AUD treatment trial, but the framework is broadly applicable to other diseases characterized by acute and chronic phases. The BIT design is built on a Bayesian probability model that links phase‐specific outcomes through the survival structure of the integrated sequence, supports interim adaptation with simulation‐calibrated futility and superiority boundaries, and accommodates prior information either as non‐informative defaults or as informative or dynamically borrowed priors when commensurate external data are available. The primary objective of the design is to identify the best integrated therapy in terms of overall survival across both phases and to formally assess its superiority over the control within a hypothesis‐testing framework; operating characteristics, including type I error and generalized power, are evaluated through simulation‐based calibration. R code implementing the design is provided.

The remainder of the paper is organized as follows. Section 2 describes the scientific background and clinical motivation for integrated therapies in AH‐AUD treatment. Section 3 introduces the notation and probability model underlying BIT. The BIT design is formally presented in Section 4. Section 5 illustrates its implementation in an AH‐AUD trial, including determination of design parameters and sample size. Simulation results are presented in Section 6. Section 7 concludes with a discussion of implications and limitations.

## 2 Integrated Therapies for AH and AUD

AH is an acute inflammatory liver condition resulting from prolonged excessive alcohol consumption. Management of AH necessarily prioritizes stabilization of the acute illness, as short‐term mortality is high. However, treatment of the acute hepatic injury alone is insufficient to ensure long‐term survival. Many patients resume alcohol consumption after symptomatic improvement, and repeated episodes of AH are associated with substantially increased risks of liver decompensation, cirrhosis, and death [29]. Multiple studies have documented high rates of return to drinking among patients diagnosed with AH, including those who have undergone liver transplantation [30, 31, 32]. A large French cohort study reported that approximately one quarter of patients resumed alcohol consumption within one year following hospitalization for AH [33], a finding supported by more recent data from the United States [32].

These observations motivate an integrated treatment strategy for AH and AUD that prioritizes management of acute liver injury followed by targeted intervention to prevent relapse to alcohol use. Conceptually, stabilization of hepatic function improves short‐term survival and creates an opportunity for patients to engage in alcohol cessation interventions. Sequentially integrating therapies across the acute and post‐acute phases is therefore hypothesized to reduce relapse risk, prevent recurrent liver injury, and improve long‐term prognosis.

The effect of such integrated AH–AUD treatment strategies on patient survival, however, has not been formally evaluated in randomized trials. Existing clinical trials in AH have focused almost exclusively on acute‐phase therapies. These include trials of pentoxifylline, a vasodilator with anti‐inflammatory properties [2], and anakinra, an interleukin‐1β antagonist [3, 34], typically compared with corticosteroids such as methylprednisolone or prednisone. Corticosteroids remain standard‐of‐care therapy for AH in patients without contraindications [35, 36, 37]. Although results across these trials have varied, none have demonstrated clear benefits of experimental therapies on intermediate‐ or long‐term survival outcomes (e.g., six‐month or one‐year survival). Notably, none of these studies incorporated interventions targeting AUD. This pattern of negative single‐component trials is informative in its own right: the long‐term clinical outcome in AH patients is determined jointly by the success of acute hepatic stabilization and the durability of post‐acute alcohol abstinence, and a trial that targets only one of these components cannot detect benefits that accrue across the integrated treatment course. A trial design tailored to the joint evaluation of acute and post‐acute therapies is therefore needed.

In parallel, multiple trials have evaluated pharmacological, behavioral, and combined pharmacological‐behavioral interventions for AUD [38, 39, 40, 41]. These studies were conducted primarily in populations with AUD but without advanced alcohol‐related liver disease. Consequently, their effects on survival among patients with AH remain largely unknown.

We consider the design of a new randomized clinical trial to test the hypothesis that an integrated treatment strategy for severe AH and AUD improves overall survival. Specifically, we compare an experimental pharmacologic therapy for acute AH (treatment B) with usual AH care (treatment A, corticosteroids), followed by a novel pharmacological–behavioral intervention for AUD (treatment D) [42, 43] versus usual care for AUD (treatment C, consisting of encouragement of abstinence and referral to substance use treatment services). The primary objective is to identify the integrated AH–AUD treatment strategy that maximizes overall survival through the end of follow‐up.

**FIGURE 1:**
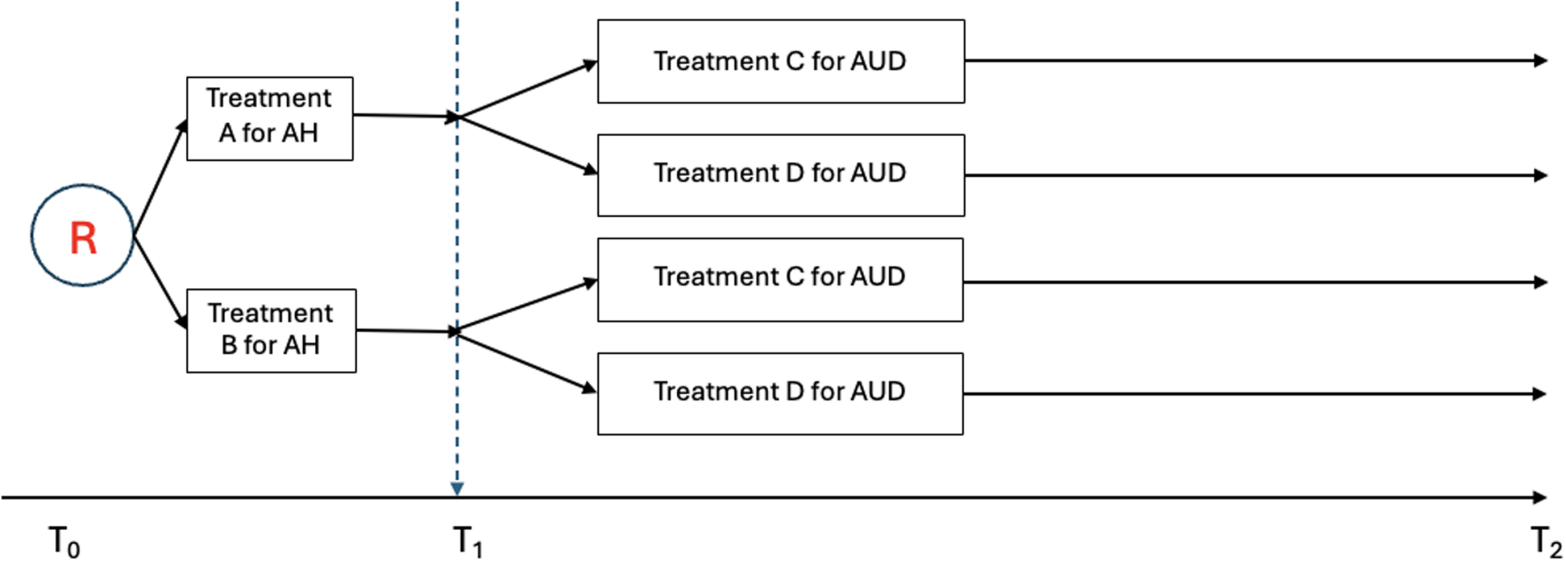
Illustration of the clinical trial for alcohol‐associated hepatitis (AH) with underlying alcohol use disorder (AUD). In this trial, treatment A represents the standard of care for AH; treatment B is the experimental pharmacological agent for AH. All participants are randomized at $t_{0}$ to receive either A or B. Survivors at $t_{1}$ either receive usual AUD care C or a novel pharmacological‐behavioral AUD intervention D.

As depicted in Figure 1, four integrated treatment combinations are under evaluation, with all comparisons benchmarked against usual care (A+C). The trial consists of two clinically distinct phases. At enrollment ($t_{0}$), all participants are randomized to one of the integrated treatment sequences. During the acute phase ($t_{0}$ to $t_{1}$), patients receive either AH treatment A or B and are followed for short‐term survival. Patients who survive to $t_{1}$ then receive the AUD component (C or D) assigned as part of their original treatment sequence and are followed through $t_{2}$. The primary estimand is the overall survival probability over the interval $\left[t_{0},t_{2}\right]$ under each integrated treatment strategy.

Although the structure of the trial resembles a 2×2 factorial design, an important distinction arises from the sequential nature of treatment delivery. In traditional factorial designs, all treatment components are administered concurrently and outcomes are observed in a single phase. In contrast, in sequentially integrated therapy trials, receipt of later‐phase treatment is conditional on survival through earlier phases. Patients who die during the acute phase do not have the opportunity to receive post‐acute therapy. As a result, overall survival is naturally expressed as a function of both phase‐specific survival probabilities and their conditional relationship. This structure motivates the use of probability models that explicitly link outcomes across phases and decision rules that operate at the level of integrated treatment sequences, rather than isolated treatment components.

More concretely, the AH‐AUD setting imposes three specific requirements on the trial design that are not met by standard multi‐arm or two‐stage designs. (1) The analysis must explicitly model both phase‐specific survival probabilities and their conditional dependence; treating each integrated sequence as a single static arm, as in a multi‐arm multi‐stage design, would discard the phase‐level information that distinguishes integrated therapies from simple combination treatments and would obscure the source of any observed treatment effect. (2) Because the order of treatments is clinically mandated rather than chosen in response to intermediate outcomes, randomization should occur at trial entry to the entire sequence rather than at the start of Phase 2 as in a SMART design; re‐randomization is biologically awkward given the fixed ordering, and conditioning on a Phase 1 survivor population that may not be exchangeable across Phase 1 arms could introduce bias into Phase 2 comparisons. (3) The difficulty of recruiting patients with severe AH and concurrent AUD [44], together with the cost of 12‐month follow‐up, favors an adaptive design with interim analyses that can drop unpromising sequences and recognize clear successes without waiting for the full planned sample size. The BIT design presented in the subsequent sections is constructed to meet each of these requirements.

The intended study population consists of individuals diagnosed with severe AH and concurrent AUD. Severe AH is defined as a Model for End‐Stage Liver Disease (MELD) score between 20 and 35 [45, 46], recent onset of jaundice (total bilirubin >3 mg/dL), and sustained heavy alcohol consumption, defined as an average intake exceeding 40 g/day or 280 g/week for women and 60 g/day or 420 g/week for men for at least six months.

This research is a response to a recent initiative from the National Institutes of Health (NIH), which have called for novel trial designs to evaluate integrated therapies for severe AH and decompensated cirrhosis (RFA AA‐24‐004 and RFA AA‐24‐005). Within the AH research community, there is growing consensus that improved integration of AH and AUD therapies is essential for achieving meaningful gains in patient outcomes [47], a position reinforced by a recent expert panel consensus statement in *Nature Reviews Gastroenterology* & *Hepatology* [6]. The method presented in this paper is intended to support the design and implementation of such trials, including power and sample size determination, flexible Bayesian models that link outcomes across disease phases, and interim and final decision‐making procedures.

## 3 The Probability Model Underlying the Design

For narrative convenience, we introduce notation and modeling assumptions in the context of the AH–AUD treatment setting described in Section 2, though the framework applies more generally to sequentially integrated therapies.

Let $a_{j}$ denote the treatment administered during Phase 1 (acute AH treatment), for j=0,1,…,J, where $a_{0}$ represents the control treatment. At enrollment time $t_{0}$, patients are randomized once to an integrated treatment sequence that includes both a Phase 1 component $a_{j}$ and a Phase 2 component, to be delivered conditional on survival. Let Y denote the time to death. A patient is said to survive Phase 1 if $Y>t_{1}$. The Phase 1 survival probability associated with treatment $a_{j}$ is therefore $p(a_{j})=\mathrm{Pr}(Y>t_{1}|a_{j})$.

Let $n_{1j}$ denote the number of patients assigned to Phase 1 treatment $a_{j}$, and let $r_{1j}$ be the number of these patients who survive through $t_{1}$. The observed Phase 1 data are denoted by ${𝒟}_{1}=\{(n_{1j},r_{1j}):j=0,1,\ldots ,J\}$.

We use a beta‐binomial model to derive the posterior distribution of $\left[p\left(a_{j}\right)|{𝒟}_{1}\right]$:

(1) $$\begin{array}{ll} p(a_{j}) & ∼\text{Beta}(0.5,0.5),\\ r_{1j}|p(a_{j}) & ∼\text{Binomial}(n_{1j},p(a_{j})),\\ \left[p(a_{j})|{𝒟}_{1}\right] & ∼\text{Beta}(0.5+r_{1j},\;0.5+n_{1j}-r_{1j}). \end{array}$$

The Beta(0.5,0.5) prior is the Jeffreys prior for the binomial parameter [48]. It is weakly informative, with effective prior sample size of one, and yields credible intervals with near‐nominal frequentist coverage in small samples [49], a relevant property for the early interim analyses. When external evidence on $p(a_{j})$ is available, this default can be replaced by an informative or dynamically borrowed prior, with corresponding re‐calibration of the design parameters; see Section 7.

Patients who survive Phase 1 proceed to Phase 2, during which treatment for AUD is delivered. Let $b_{k}$ denote the Phase 2 treatment, for k=0,1,…,K, where $b_{0}$ represents usual care. Let $d_{jk}=(a_{j},b_{k})$ be the integrated treatment sequence consisting of Phase 1 treatment $a_{j}$ followed by Phase 2 treatment $b_{k}$. Importantly, the Phase 2 component $b_{k}$ is pre‐assigned at randomization but is delivered only if the patient survives to $t_{1}$. Let $n_{2jk}$ denote the number of patients who survive Phase 1 and receive Phase 2 treatment under regime $d_{jk}$. For the ith patient, $y_{ijk}$ is the observed survival time from $t_{1}$, subject to independent right censoring. For the AH‐AUD integrated treatment trial, the sequentially integrated treatments are detailed in Table 1.

**TABLE 1:** Sequentially integrated treatments considered for the AH trial.

Regime	Phase 1 ($a_{j}$)	Phase 2 ($b_{k}$)
$d_{00}$	$a_{0}$: corticosteroids (control)	$b_{0}$: usual AUD care
$d_{01}$	$a_{0}$: corticosteroids (control)	$b_{1}$: novel AUD therapy
$d_{10}$	$a_{1}$: experimental AH therapy	$b_{0}$: usual AUD care
$d_{11}$	$a_{1}$: experimental AH therapy	$b_{1}$: novel AUD therapy

We model Phase 2 survival using a piecewise exponential (PE) model. The interval $\left(t_{1},t_{2}\right]$ is partitioned into L disjoint subintervals $\left(s_{l-1},s_{l}\right]$, l=1,…,L, with $s_{0}\equiv t_{1}$ and $s_{L}\equiv t_{2}$. Within each subinterval, the hazard is assumed constant. Let $\lambda_{jkl}$ denote the hazard in interval $\left(s_{l-1},s_{l}\right]$ for patients receiving integrated treatment $d_{jk}$.

Let $e_{ijkl}=1$ if patient i receiving treatment $d_{jk}$ dies in interval $\left(s_{l-1},s_{l}\right]$, and $e_{ijkl}=0$ otherwise. Let $v_{ijkl}$ denote the amount of time patient i is at risk in that interval:

$$v_{ijkl}=\left\{\begin{array}{ll} s_{l}-s_{l-1},\; & \text{if}\;y_{ijk}>s_{l},\\ y_{ijk}-s_{l-1},\; & \text{if}\;y_{ijk}\in (s_{l-1},s_{l}],\\ 0,\; & \text{otherwise}. \end{array}\right.$$

Let ${𝒟}_{2}=\{(v_{ijkl},e_{ijkl}):i=1,\ldots ,n_{2jk};\;j=0,\ldots ,J;\;k=0,\ldots ,K;\;l=1,\ldots ,L\}$ denote the Phase 2 data, and let $\Theta_{2}=\{\lambda_{jkl}\}$ denote the corresponding hazard parameters. The Phase 2 likelihood, conditional on Phase 1 survival, is given by

(2) $$L({𝒟}_{2}|{𝒟}_{1},\Theta_{2})=\overset{J}{\underset{j=0}{\prod }}\overset{K}{\underset{k=0}{\prod }}\overset{n_{2jk}}{\underset{i=1}{\prod }}\overset{L}{\underset{l=1}{\prod }}\lambda_{jkl}^{e_{ijkl}}\exp(-\lambda_{jkl}v_{ijkl}).$$

We assign independent Gamma(0.01,0.01) priors to each $\lambda_{jkl}$, corresponding to weakly informative priors commonly used in Bayesian survival analysis [50]. Let $𝒟=({𝒟}_{1},{𝒟}_{2})$. The resulting posterior distribution is

(3) $$\left[\lambda_{jkl}|𝒟\right]∼\text{Gamma}\left(0.01+\overset{n_{2jk}}{\underset{i=1}{\sum }}e_{ijkl},\;0.01+\overset{n_{2jk}}{\underset{i=1}{\sum }}v_{ijkl}\right).$$

Let $\pi (d_{jk})$ denote the overall survival probability through the end of Phase 2 for patients assigned to integrated treatment $d_{jk}$, that is, $\pi (d_{jk})=\mathrm{Pr}(Y>t_{2}|d_{jk})$. Under the proposed model, this quantity can be expressed as

$$\begin{array}{ll} \pi (d_{jk}) & =\mathrm{Pr}(Y>t_{1}|a_{j})\;\mathrm{Pr}(Y>t_{2}|Y>t_{1},d_{jk})\\ & =p(a_{j})\;\exp\left\{-\overset{L}{\underset{l=1}{\sum }}\lambda_{jkl}(s_{l}-s_{l-1})\right\}. \end{array}$$

Posterior inference for $\pi (d_{jk})$ is obtained by sampling from the joint posterior distribution of $\left\{p(a_{j}),\lambda_{jkl}\right\}$ via Gibbs sampling, using Equations ((1), (2), (3)).

## 4 The Design

### 4.1 Decision Rules for Treatment Optimization

Under the BIT framework, there are (J+1)×(K+1) possible integrated treatment sequences under investigation. Let $d_{00}$ be the control treatment, consisting of usual care for AH in Phase 1 followed by usual care for AUD in Phase 2. The objective of the design is to identify the integrated treatment sequence that yields the highest overall survival probability through the end of Phase 2, that is, over the interval $\left[t_{0},t_{2}\right]$, relative to the control.

Let Δ>0 denote a prespecified minimal clinically meaningful difference in overall survival. For each integrated treatment sequence $d_{jk}$, with (j,k)≠(0,0), we compute the posterior probability that its overall survival probability exceeds that of the control by at least Δ:

(4) $$PP(d_{jk})=\mathrm{Pr}\left\{\pi (d_{jk})>\pi (d_{00})+\Delta |𝒟\right\}.$$

The quantity $PP(d_{jk})$ represents the posterior probability that the integrated treatment sequence $d_{jk}$ provides a clinically meaningful improvement in overall survival relative to usual care. These posterior probabilities form the primary basis for treatment optimization in the BIT design.

At each interim or final analyses, integrated treatment sequences are ranked according to their posterior probabilities $PP(d_{jk})$. The sequence with the largest posterior probability is considered the leading candidate for selection. In practice, posterior probabilities for competing sequences may be similar, particularly at early interim analyses when information is limited. To ensure stability of the decision process, if the difference between the largest and second‐largest posterior probabilities is smaller than a prespecified tolerance ε (e.g., ε=0.01), no definitive selection is made at that analysis. In such cases, both sequences are retained for continued evaluation. At the final analysis, ties or near‐ties may be resolved by selecting the sequence with the larger posterior mean of $\pi (d_{jk})$.

The decision rules based on $PP(d_{jk})$ are heuristic Bayesian rules whose frequentist operating characteristics, including error rates and power, are evaluated through simulation under a range of scenarios, as described in Section 6.

### 4.2 Trial Implementation

In the proposed BIT design, each patient is randomized only once at trial entry. The assigned treatment sequence, including both Phase 1 and Phase 2 components, is fixed at randomization, with the Phase 2 component delivered only if the patient survives to $t_{1}$. An alternative approach would involve two‐stage randomization, in which the Phase 2 treatment is re‐randomized among survivors at $t_{1}$. Here we adopt single randomization to ensure balanced allocation across integrated treatment sequences throughout the trial. Because the candidate set $C_{r}$, which consists of therapeutic combinations that have not been dropped for futility relative to the control, is adaptively updated, a two‐stage randomization scheme could induce imbalanced allocation among remaining treatment sequences after exclusions. Single randomization avoids this issue and preserves fair allocation among candidate integrated therapies at all stages of the trial. Beyond this implementation consideration, single randomization also simplifies identification of the joint estimand $\pi (d_{jk})$ and avoids selection on Phase 1 survival when comparing integrated sequences. In each interim analysis, we simply compare $PP(d_{jk})$ with the lower boundary and construct the candidate set.

Before presenting the formal specification, we summarize the BIT procedure in plain terms. Patients are enrolled continuously and randomized with equal probability to one of the (J+1)(K+1) integrated treatment sequences. At each of the R prespecified interim analyses, the posterior probability that a non‐control sequence exceeds the control by the clinically meaningful margin Δ is computed from accumulated data on patients with complete follow‐up. Sequences whose posterior probability falls below a futility boundary are dropped from the candidate set, and subsequent enrollees are randomized only among the control and the remaining candidates. If the leading sequence exceeds a superiority boundary at any interim, the trial may stop early and that sequence is declared optimal. Otherwise, the trial continues to its planned maximum sample size, and at the final analysis the integrated sequence with the largest posterior probability above δ is selected as the optimal therapy. The full procedure is summarized in Algorithm 1.

ALGORITHM 1 **BIT design implementation.**

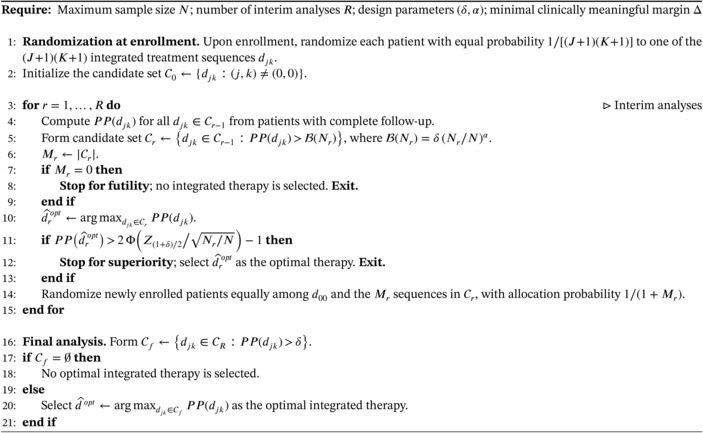

**FIGURE 2:**
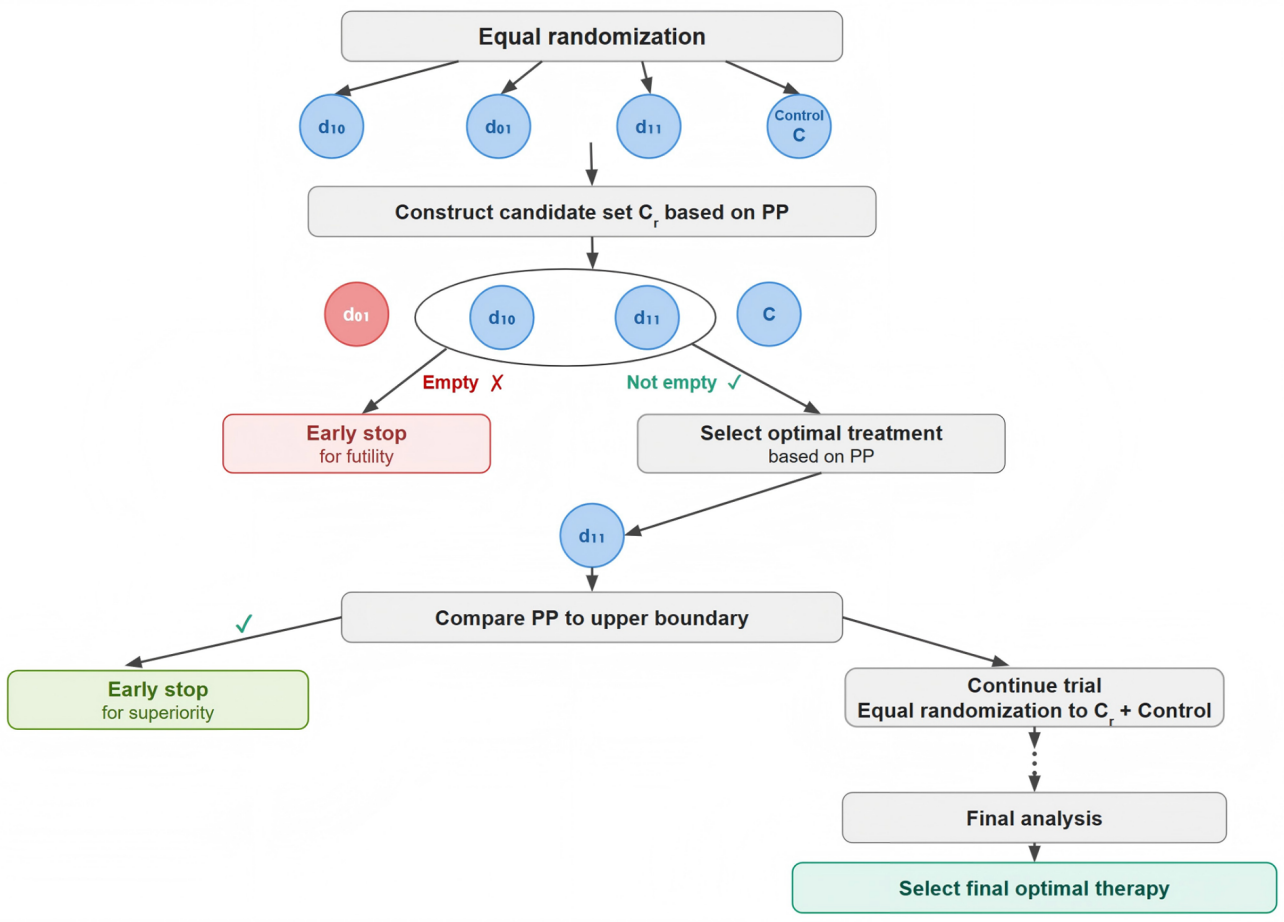
Flow diagram of the steps for BIT implementation.

A flow diagram is included to illustrate the key steps; see Figure 2. Key steps are explained below:

1. Upon enrollment, each patient is randomized with equal probability to one of the (J+1)(K+1) integrated treatment sequences $d_{jk}$, with randomization ratio 1/[(J+1)(K+1)].
2. For the rth interim analysis, r=1,…,R, compute posterior probabilities $PP(d_{jk})$ based on all accumulated data and construct the candidate set
(5) $${𝒞}_{r}=\left\{d_{jk}:PP(d_{jk})>ℬ(N_{r})\right\},\;(j,k)\neq (0,0),$$
  where $ℬ(N_{r})$ is a lower boundary for posterior probabilities that serves to eliminate treatment sequences with insufficient evidence of efficacy. The candidate set therefore contains all treatment combinations whose posterior probability is above the boundary for elimination for lack of efficacy. It should be noted that the decision rule $PP(d_{jk})$ is one‐sided by construction. It is a posterior probability analogue of a group‐sequential boundary for a one‐sided test.
  The boundary $ℬ(N_{r})$ is specified as a function of the cumulative sample size at the rth interim analysis. While any monotone increasing function could be used in principle, we adopt the boundary proposed in the Bayesian Optimal Phase II (BOP2) design [16],
$$ℬ(N_{r})=\delta {\left(\frac{N_{r}}{N}\right)}^{\alpha },$$
  where N is the planned maximum sample size and δ,α∈(0,1) are design parameters. This boundary has been shown empirically to provide desirable trade‐offs between early elimination of ineffective treatments and retention of promising candidates in a range of Bayesian adaptive trial settings [15, 16]. In the present context, its operating characteristics, including type I error, power, and expected sample size, are calibrated via simulation.
3. Let $M_{r}=|{𝒞}_{r}|$ denote the number of treatment sequences in the candidate set. If $M_{r}=0$, the trial is terminated early for futility, and no integrated therapy is selected.
  If $M_{r}>0$, define the leading candidate at the rth interim analysis as
$${\hat{d}}_{r}^{\;opt}=\arg\underset{d_{jk}\in {𝒞}_{r}}{\max}PP(d_{jk}).$$
  To allow for early termination for superiority, we compare $PP({\hat{d}}_{r}^{\;opt})$ to an upper boundary. Specifically, following the approach used in the two‐arm BOP2 design [15], we declare early superiority if
$$PP({\hat{d}}_{r}^{\;opt})>2\Phi \;\left(\frac{Z_{(1+\delta )/2}}{\sqrt{N_{r}/N}}\right)-1,$$
  where Φ(·) is the standard normal cumulative distribution function and $Z_{q}$ denotes the qth quantile of the standard normal distribution. This criterion may be viewed as a posterior‐probability analogue of a conservative group‐sequential superiority rule. Its operating characteristics depend on the prior specification and are therefore assessed through simulation rather than derived analytically.
  If the superiority criterion is met, the trial is terminated early and ${\hat{d}}_{r}^{\;opt}$ is selected as the optimal integrated therapy. Otherwise, newly enrolled patients are randomized equally among the control treatment $d_{00}$ and the $M_{r}$ candidate treatments in ${𝒞}_{r}$, with allocation probability $1/(1+M_{r})$.
  Patients already enrolled on an integrated therapy that is excluded at an interim analysis continue on their originally assigned therapy by default, with follow‐up maintained as planned. However, outcomes observed after exclusion are not included in subsequent analyses for the excluded therapy. If clinically warranted, investigators may instead transition such patients to an active alternative for ethical reasons; in that case, follow‐up under the originally assigned therapy is censored at transition, and outcomes observed after transition are not included in subsequent analyses of either therapy.
4. Steps 2 and 3 are repeated until either early termination occurs or the final interim analysis (r=R) is reached.
5. At the final analysis, the candidate set is defined as ${𝒞}_{f}=\left\{d_{jk}:PP(d_{jk})>\delta \right\}$. If ${𝒞}_{f}$ is empty, no optimal integrated therapy is selected. Otherwise, the final selected treatment is
$${\hat{d}}^{\;opt}=\arg\underset{d_{jk}\in {𝒞}_{f}}{\max}PP(d_{jk}).$$

The late‐onset outcomes require additional consideration for trial implementation. At the time of interim analyses, some patients may still be under follow‐up with their survival outcomes not yet observed. A naïve approach that classifies these patients as survivors can inflate the estimated treatment effect and introduce bias into the analysis. To address this issue, the BIT design restricts interim analyses to patients with complete follow‐up, thereby ensuring statistical rigor. It is worth noting that late‐onset outcomes do not pose a concern for the final analysis, as it is conducted only after all patients have completed follow‐up.

The BIT design involves two tuning parameters, (δ,α), which govern interim elimination and early stopping behavior. These parameters are selected through simulation‐based calibration to control the type I error while maximizing power. Specifically, we evaluate the probability of incorrectly selecting an integrated therapy when all candidate therapies are ineffective relative to the control. This quantity corresponds to the probability of false selection under the global null and serves as the primary error metric for design calibration. Parameter values (δ,α) that achieve the desired error rate are identified, and among those, values that maximize power are selected.

To assess power, we consider two generalized power measures. GP1 is defined as the probability that the selected integrated therapy ${\hat{d}}^{\;opt}$ is the truly optimal therapy. GP2 is defined as the probability that ${\hat{d}}^{\;opt}$ is superior to the control, regardless of whether it is globally optimal. GP1 is therefore a stricter criterion than GP2. The choice of power metric depends on the clinical context and the relative consequences of false negative versus suboptimal selections.

All design parameters and operating characteristics are evaluated through simulation. Computational details and R code implementing the BIT design are provided in the Supporting Information.

## 5 Example: Designing an AH‐AUD Trial

We illustrate the proposed BIT design using a hypothetical randomized trial for integrated treatment of AH and AUD. The example demonstrates the determination of sample size and calibration of design parameters through simulation‐based grid search. Because justification of sample size and operating characteristics is a required component of clinical trial protocols, we present these elements explicitly within the BIT framework.

Patients in the acute stage are followed for $t_{1}=1$ month. Those who survive to the end of the acute AH phase then enter the AUD phase and are followed until $t_{2}=12$ months. We consider a design with R=2 interim analyses. The first interim analysis is conducted when approximately 30% of enrolled participants have completed follow‐up through Phase 2, and the second when approximately 70% have completed Phase 2 follow‐up. As noted in the previous section, patients with pending outcomes are excluded from the interim analysis. The minimal clinically meaningful difference in overall survival is set to Δ=0.05. Two data‐generating scenarios are considered, representing null and alternative hypotheses for the Phase 1 survival probabilities $p(a_{j})$ and overall survival probabilities $\pi (d_{jk})$. These scenarios are summarized in Table 2.

**TABLE 2:** Overall survival probabilities associated with each treatment combination in the six simulated scenarios. Here, $p(a_{0})$ and $p(a_{1})$ are the true probability of a participant surviving Phase 1 of the study, and $\pi (d_{jk})$ is the overall survival probability associated with treatment combination $d_{jk}$ for both phases of the trial. The type I error is to be evaluated at values in the red font. GP1 is to be evaluated at values in the blue font. GP2 is to be evaluated at values in green or blue fonts.

Scenarios	$p\left(a_{0}\right)$	$p\left(a_{1}\right)$	$\pi \left(d_{00}\right)$	$\pi \left(d_{01}\right)$	$\pi \left(d_{10}\right)$	$\pi \left(d_{11}\right)$
1	0.7	0.7	0.50	0.50	0.55	0.55
2	0.7	0.8	0.50	0.50	0.55	0.70
3	0.7	0.8	0.50	0.65	0.55	0.70
4	0.7	0.8	0.50	0.65	0.65	0.70
5	0.7	0.8	0.50	0.50	0.50	0.50
6	0.8	0.7	0.50	0.60	0.65	0.65

The timing of interim analyses is an important practical consideration in adaptive trial design. Interim analyses conducted too early may be based on insufficient information, while analyses conducted too late may limit the potential efficiency gains of adaptation. In general, we recommend scheduling the first interim analysis when approximately 25%–50% of participants have completed follow‐up for the primary endpoint, depending on expected event rates and accrual speed.

At the time of each interim analysis, some previously enrolled participants may still be under follow‐up with Phase 2 outcomes pending. In this example, interim analyses are based only on participants with fully observed outcomes through $t_{2}$. Patients with incomplete follow‐up are excluded from interim calculations. However, if their corresponding treatment sequences remain active through the end of the trial, these patients are included in the final analysis once all outcomes have been observed. This approach simplifies implementation and avoids assumptions about partially observed survival data, while preserving the validity of the final analysis.

In BIT, once a treatment combination is excluded from the candidate set at an interim analysis, it remains excluded at all subsequent interim and final analyses, so the candidate set $C_{r}$ narrows monotonically in the trial. This preclude the possibility that the final analysis contradicts the interim results, because excluded treatment are not re‐evaluated.

Power is evaluated using the generalized power metric GP2, defined as the probability that the selected integrated therapy is superior to the control, regardless of whether it is globally optimal. The target one‐sided error and power levels are set to 5% under Scenario 1 (null scenario) and 80% GP2 under Scenario 2 (alternative scenario), respectively.

In simulation, Phase 2 survival times are generated from Weibull distributions, with hazard ratios chosen to match the specified values of $p(a_{j})$ and $\pi (d_{jk})$. We assume that approximately 60% of deaths among patients surviving Phase 1 occur during the first half of Phase 2 follow‐up. For model fitting, the Phase 2 follow‐up window is partitioned into two equal‐length intervals, (1,6.5] and (6.5,12], consistent with the piecewise exponential model described in Section 3. Sensitivity analyses examining alternative interval partitions are reported in Section 6.

Sample size determination proceeds via grid search. We begin with a total sample size of 150, which represents a viable enrollment target as shown in a prior trial by the same consortium [34]. We conduct simulation studies to estimate the empirical false selection probability under Scenario 1 and GP2 under Scenario 2 across a grid of design parameters (α,δ). For each configuration, 5000 simulated trials are generated. If no combination of (α,δ) simultaneously satisfies the target error and power criteria, the total sample size is increased in increments of 10 and the procedure is repeated. The smallest sample size for which at least one admissible parameter pair exists is selected. When multiple admissible (α,δ) pairs are available for a given sample size, the pair yielding the highest GP2 is chosen.

Results of the calibration procedure are summarized in Table 3. A total sample size of 300 participants, with design parameters α=0.98 and δ=0.95, achieves an empirical false selection probability (type I error) of 4.86% under Scenario 1 and a GP2 of 81.86% under Scenario 2. No smaller sample size satisfied both criteria. Accordingly, a total sample size of 300 is selected for the trial.

**TABLE 3:** Sample size determination for example AH‐AUD treatment trial: type I error rate and generalized power (GP2) under design parameters optimized with a sample size of 300. The corresponding design parameters are α=0.98 and δ=0.95.

	δ							
	Type I error for Scenario 1 (%)				GP2 for Scenario 2 (%)			
α	0.94	0.95	0.96	0.97	0.94	0.95	0.96	0.97
0.88	7.00	5.56	4.30	3.04	83.76	81.84	79.30	75.06
0.90	7.54	5.78	4.48	3.76	83.78	81.50	78.68	75.00
0.92	7.52	6.40	4.40	3.22	83.44	81.84	78.70	73.98
0.94	6.90	6.42	4.46	3.16	83.08	81.06	78.88	74.80
0.96	6.86	5.66	4.18	3.42	83.96	81.70	79.12	75.76
0.98	6.88	4.86	4.24	3.02	82.96	81.86	79.22	75.44

**FIGURE 3:**
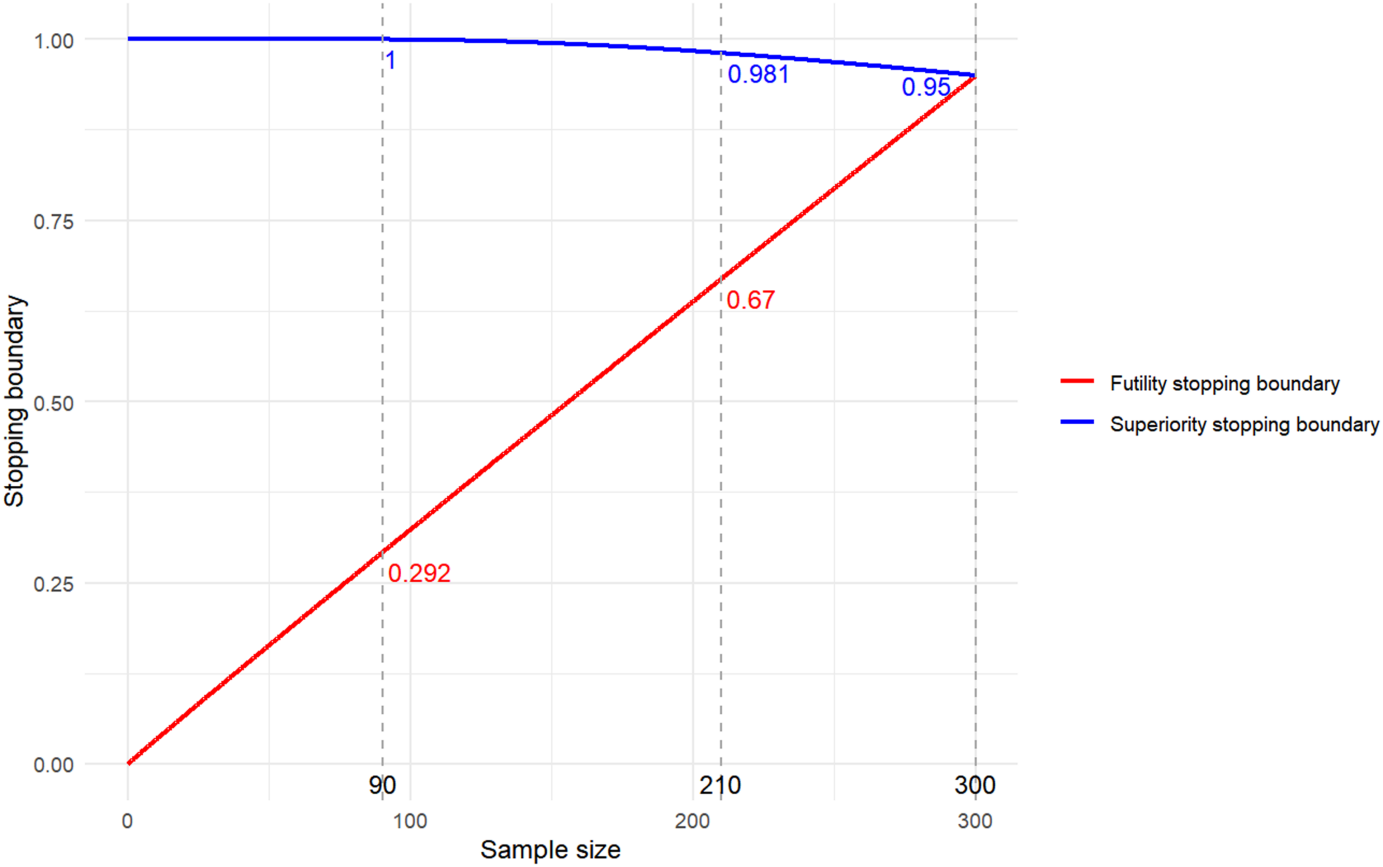
Futility and superiority stopping boundaries for interim analyses in the example AH‐AUD trial with BIT design.

Interim decisions are based on posterior probabilities

$$PP(d_{jk})=\mathrm{Pr}\;\left\{\pi (d_{jk})>\pi (d_{00})+\Delta |𝒟\right\},$$

computed using cumulative data available at each interim analysis. In this example, we set Δ=0.05. Decisions on discontinuing the trial for superiority or futility can also be made by comparing the estimated $PP\left(d_{jk}\right)$ with the corresponding upper and lower stopping boundaries in Figure 3. The superiority stopping boundaries, implied by the calibrated design parameters α and δ, are 1.00 and 0.981 at the first and second interim analyses, indicating that no integrated treatment is selected as superior during the first interim analysis. The corresponding futility boundaries are 0.292 and 0.67. At the final analysis, integrated treatment sequences with $PP(d_{jk})>\delta =0.95$ constitute the candidate set from which the optimal treatment is selected.

## 6 Simulation Studies

### 6.1 Design Setting and Data Generation

Simulation studies were conducted to evaluate the operating characteristics of the proposed BIT design under a range of clinically plausible scenarios. Six data‐generating scenarios were considered, each defined by different combinations of Phase 1 survival probabilities $p(a_{j})$ and overall survival probabilities $\pi (d_{jk})$, as summarized in Table 2. These scenarios were selected to represent settings under the global null, partial alternatives, and global alternatives.

Scenarios 1 and 5 represent global null settings in which none of the experimental integrated therapies outperform the control treatment $d_{00}$. In Scenario 1, Phase 1 survival probabilities are equal across treatments, $p(a_{1})=p(a_{0})$, whereas in Scenario 5 the experimental Phase 1 treatment improves short‐term survival, $p(a_{1})>p(a_{0})$, but does not lead to improved overall survival when combined with Phase 2 therapies. Scenario 2 represents a simple alternative in which $d_{11}$ is the only effective integrated treatment. Scenario 3 introduces an additional effective treatment $d_{01}$, although $d_{11}$ remains optimal. Scenarios 4 and 6 represent global alternative settings in which multiple integrated therapies are effective. In Scenario 4, $p(a_{1})>p(a_{0})$ and $d_{11}$ yields the highest overall survival probability (0.70), while $d_{01}$ and $d_{10}$ are also effective with overall survival probabilities of 0.65. In Scenario 6, two integrated treatments, $d_{10}$ and $d_{11}$, are equally optimal, each yielding an overall survival probability of 0.65, while $p(a_{1})<p(a_{0})$.

Phase 1 survival outcomes were generated from a Bernoulli distribution $\text{Bernoulli}\{p(a_{j})\}$. For patients surviving Phase 1, Phase 2 time‐to‐death outcomes were generated from Weibull distributions as described in Section 5, with parameters chosen to match the specified overall survival probabilities. Enrollment times were generated from an exponential distribution corresponding to a mean accrual rate of approximately 9 patients per month across all sites, reflecting the relatively slow recruitment typically observed in AH clinical trials [44]. Unless otherwise stated, all remaining design parameters, including total sample size, calibrated values of (δ,α), and the number and timing of interim analyses, were set to the values determined in Section 5.

To provide context for the performance of the proposed design, we considered three comparator designs. The first was a fixed (non‐adaptive) design that evaluates all integrated treatment combinations in a single final analysis without interim monitoring. This design is structurally similar to a factorial trial, but extends beyond the factorial framework by explicitly modeling the sequential nature of treatment delivery or conditional survival. The second and third comparator designs incorporated interim analyses but allowed early stopping for futility only (FS) or superiority only (SS), respectively. These comparators isolate the contributions of futility monitoring and superiority monitoring to overall trial performance. Because no existing designs directly accommodate sequentially integrated therapies with adaptive arm elimination and regime‐level decision rules, comparisons were limited to designs with closely related features.

We evaluated the performance of the proposed design using four metrics based on 5000 simulated trials:

1. the probability of false selection under the global null;
2. generalized power (GP1 and GP2);
3. average sample size; and
4. the probability of early stopping.

### 6.2 Simulation Results

Results are summarized in Tables 4 and 5. Table 4 reports the false selection probability and generalized power measures, while Table 5 reports average sample size and early stopping probabilities.

**TABLE 4:** Simulation results for type I error, generalized power (GP) 1 and 2, among the BIT, fixed, the stopping for futility only (FS), and the stopping for superiority only (SS) designs.

Scenario	Type I error (%)				GP1 (%)				GP2 (%)			
	BIT	Fixed	FS Only	SS Only	BIT	Fixed	FS Only	SS Only	BIT	Fixed	FS Only	SS Only
1	4.86	5.50	4.84	5.18								
2	0.32	0.16	0.20	0.32	81.68	61.30	81.18	76.46	81.68	61.30	81.18	76.46
3	0.18	0.10	0.02	0.08	47.90	41.20	48.20	49.58	94.00	77.10	92.88	92.00
4					41.82	37.86	43.94	43.12	92.82	78.72	93.02	92.66
5	2.44	1.3	1.22	2.62								
6					36.28	38.32	33.06	35.88	46.48	44.86	44.18	47.18

**TABLE 5:** Simulation results for the average sample size and early stopping probability among the BIT, the stopping for futility only (FS), and the stopping for superiority only (SS) designs.

Scenario	Average sample size			Early stopping probability (%)				
	BIT	FS Only	SS Only	BIT			FS Only	SS Only
				FS	SS	Total		
1	226.46	212.62	298.26	58.96	1.64	60.60	67.54	1.78
2	247.77	288.07	257.42	5.76	47.24	53.00	8.14	44.88
3	225.24	296.94	229.31	1.18	69.20	70.38	2.16	66.88
4	226.25	297.72	229.40	1.16	68.66	69.82	1.70	67.54
5	212.48	195.07	298.97	71.42	0.98	72.40	78.30	1.08
6	261.93	273.83	281.01	15.32	21.10	36.42	20.70	20.32

Table 4 shows that all designs under consideration maintain the false selection probability close to the nominal 5% level under global null scenarios. In terms of power, when only a single effective integrated therapy exists, the BIT and futility‐only (FS Only) designs consistently outperform the superiority‐only (SS Only) and non‐adaptive comparator designs. When multiple effective integrated therapies are present, the BIT, FS Only, and SS Only designs exhibit comparable generalized power, while the non‐adaptive comparator remains the least efficient.

For example, in Scenario 2, where $d_{11}$ is the only effective treatment and GP1 and GP2 coincide, the BIT and FS Only designs achieve approximately 5% higher power than the SS Only design and approximately 20% higher power than the non‐adaptive comparator. In Scenario 3, where both $d_{01}$ and $d_{11}$ are effective and $d_{11}$ is optimal, the BIT, FS Only, and SS Only designs yield similar power levels. These power levels are approximately 6%–8% higher than the non‐adaptive comparator for GP1 and 15%–17% higher for GP2.

Table 5 summarizes average sample sizes and early stopping probabilities. The non‐adaptive comparator design does not allow early stopping and therefore always reaches the maximum sample size of 300. In contrast, the BIT design consistently yields smaller average sample sizes due to its ability to terminate early for either futility or superiority. The performance of the FS Only and SS Only designs depends strongly on the underlying scenario.

Under global null scenarios (e.g., Scenario 1), the FS Only design stops early for futility in 67.54% of simulated trials, resulting in an average sample size of 212.62, closely matching the performance of the BIT design. The SS Only design, which lacks futility stopping, rarely terminates early in this setting and has an average sample size of 298.26. Conversely, under scenarios in which all experimental integrated therapies are effective (e.g., Scenario 4), the SS Only design frequently stops early for superiority, with an early stopping probability of 67.54 and an average sample size of 229.4, comparable to the BIT design. In this setting, the FS Only design loses efficiency and typically reaches near‐maximal sample size.

Additional simulation studies assessed the sensitivity of the BIT design to key modeling assumptions. Operating characteristics were stable under alternative prior specifications, Beta(1,1) for $p(a_{j})$ and Gamma(0.1,0.1) for $\lambda_{jkl}$ (Supporting Information: Table S4); under increases in the number of piecewise exponential intervals from L=2 to L=3 and L=4 (Supporting Information: Table S5); under alternative partition points placed at one‐third or two‐thirds of the Phase 2 follow‐up window rather than the midpoint (Supporting Information: Table S6); and under replacement of the piecewise exponential fitting model with a Weibull model (Supporting Information: Table S7). Across these specifications, the empirical false selection probability remained within roughly one percentage point of the nominal level and generalized power varied by no more than two percentage points relative to the default configuration.

Overall, the simulation results demonstrate that the BIT design provides a favorable balance between error control, power, and sample size efficiency. Relative to comparator designs, BIT achieves higher or comparable generalized power while consistently reducing average sample size through flexible early stopping. These properties suggest that the BIT design offers a practical and robust approach for evaluating sequentially integrated therapies.

## 7 Discussion

Integrated treatment strategies are increasingly recognized as essential for addressing complex diseases in which patients transition from acute illness to long‐term management of an underlying chronic condition. In such settings, clinical outcomes are rarely determined by a single therapeutic component. In AH, short‐term stabilization of acute liver injury must be followed by sustained treatment of AUD to prevent relapse and improve long‐term prognosis [6, 29]. Despite the clinical relevance of such care pathways, practical trial designs that explicitly evaluate sequentially integrated therapies remain limited.

In this paper, we propose the Bayesian adaptive design for sequential Integrated Therapies (BIT), a flexible design framework tailored to settings in which treatments are delivered sequentially across clinically distinct phases and outcomes are defined over the entire treatment sequence. The BIT design formalizes overall survival as the primary estimand and bases treatment selection on posterior probabilities comparing integrated treatment sequences with a control. This regime‐level perspective aligns naturally with clinical decision‐making and facilitates transparent communication of treatment effectiveness.

The BIT design combines elements from existing Bayesian adaptive methodologies while extending them to accommodate sequentially integrated therapies. As detailed in Section 1, BIT differs from SMART and dynamic treatment regime designs in targeting a fixed integrated sequence rather than a conditional decision rule, and from multi‐arm multi‐stage designs in modeling phase‐level survival rather than treating each integrated sequence as a static arm. It supports incorporation of prior information through Bayesian priors, enabling the use of existing evidence to improve efficiency in accordance with recent regulatory guidance [51]. Interim analyses allow early termination for futility or superiority, improving resource utilization without compromising final inference. Importantly, the operating characteristics of the design, including the probability of false selection and generalized power, are calibrated and evaluated through simulation. Our results demonstrate that under appropriate choices of design parameters, the design can achieve desirable trade‐offs between error control, power, and sample size efficiency across a range of plausible clinical scenarios.

As with most Bayesian adaptive designs, the stopping boundaries are calibrated via simulation to control the type I error rate and achieve desirable power. In this paper, we adopt the same functional form for the stopping boundaries as in the BOP2 design for randomized trials [15]. Alternatively, other functional forms may be used, provided they satisfy the following conditions: (1) the futility boundary is a monotonically increasing function of the information fraction; (2) the superiority boundary is a monotonically decreasing function of the information fraction; and (3) the two boundaries converge to a single point at the final analysis.

While the BIT design was motivated by the AH–AUD setting, its applicability extends to other disease contexts involving sequential treatment phases. Examples include oncologic care pathways in which radiotherapy is followed by immunotherapy [7, 52], treatment of acute sexually transmitted infections followed by partner notification and behavioral interventions [9], and hospital management of decompensated heart failure followed by outpatient cardiac rehabilitation [8]. In these settings, later‐phase outcomes are conditional on survival or response to earlier treatment, making integrated sequence‐level evaluation particularly relevant. The design is also well aligned with regulatory interest in Bayesian adaptive approaches that are supported by rigorous simulation‐based justification [53].

The BIT framework can accommodate alternative outcome types in either phase with relatively straightforward modifications. The Beta‐Binomial model for Phase 1 accommodates any binary endpoint without structural change; plausible alternatives to short‐term survival in liver disease trials include early biochemical response to corticosteroid therapy, need for liver transplantation, hepatic decompensation, or composite endpoints such as major adverse liver outcomes. When the Phase 1 follow‐up window is long enough that the timing of death carries additional information, the Beta‐Binomial model can be replaced by a parametric survival model structurally analogous to the Phase 2 model in Equations (2) and (3), with the joint estimand redefined accordingly. For Phase 2, the piecewise exponential model can be replaced with a normal or hierarchical normal model for continuous endpoints such as quality of life, a proportional odds model for ordinal endpoints, or a win‐ratio formulation for hierarchical composite endpoints. In each case the remaining design components, such as decision quantity $PP(d_{jk})$, simulation‐based calibration of (α,δ), and adaptive candidate‐set update, carry over directly.

Several potential extensions and limitations merit discussion. First, although we model survival across phases using a conditional factorization, more complex multi‐state or illness‐death models could be incorporated to explicitly represent intermediate events such as relapse or rescue therapy. Second, interim analyses in the current implementation exclude participants with incomplete follow‐up; alternative approaches that incorporate partial information from censored observations could further improve efficiency and warrant future investigation. Third, although the current work focuses on efficacy endpoints, the framework could be extended to jointly consider safety outcomes or composite benefit‐risk criteria. Last but not least, we adopt non‐informative priors as default choices in this paper, specifically Beta(0.5,0.5) for $p(a_{j})$ and Gamma(0.01,0.01) for $\lambda_{jkl}$. When reliable external data are available, informative priors can be considered. For example, the short‐term effects of corticosteroids on 30‐ and 90‐day survival in alcoholic hepatitis (AH) are well documented [3, 34]. However, fully informative prior approaches based on external data can be risky and may bias statistical inference when the external data are not commensurate with the trial data. As recommended in the FDA's latest guidance on Bayesian methods in clinical trials [54], Bayesian dynamic information borrowing approaches, such as the elastic prior [55] or self‐adapting mixture prior [56], should be used to address prior‐data conflict and to balance efficiency with bias control. When informative or dynamically borrowed priors are adopted, re‐calibration of the design parameters (α,δ) by simulation under the chosen prior is part of the recommended workflow, since the operating characteristics established under the default priors are not invariant to the prior specification.

In summary, building on existing Bayesian adaptive design methodology [16, 21, 26, 50], we introduce the BIT framework as a practical and flexible approach for evaluating sequentially integrated therapies. By targeting clinically meaningful estimands, supporting adaptive decision‐making, and relying on simulation‐calibrated operating characteristics, the BIT design offers a useful tool for designing trials that better reflect modern, patient‐centered care.

## Computational Details

See Supporting Information, Sections 2 and 3, for computation details, including the R code for simulation implementation.

## Funding

This work was supported by the National Institutes of Health (Grant Nos. 5U24AA026969, 5R01GM150808, P30CA082709).

## Conflicts of Interest

Wanzhu Tu is a member of the Hepatology editorial board. Other authors declare no conflicts of interest.

## Supporting information




**Data S1:** Supporting Information.

## Data Availability

Data sharing not applicable to this article as no datasets were generated or analysed during the current study.
