# Supplementary material for: A Bayesian Optimal Adaptive Clinical Trial Design for Sequentially Integrated Therapies
Source: Stat Med. 2026 Jul 30;45(18-19):e70684. doi: 10.1002/sim.70684 (PMC13422262; doi:10.1002/sim.70684)
Supplement: Supplementary file 1 — Data S1: Supporting Information. [file SIM-45-0-s001.pdf]

Supplementary Material: A Bayesian Optimal Adaptive Clinical Trial Design for Sequentially Integrated Therapies

Li, Guo, Gawrieh, Shen, Zang, and Tu

This document contains supplementary material to the manuscript titled "A Bayesian Optimal Adaptive Clinical Trial Design for Integrated Therapies."

This supplement has three sections: (1) Additional simulation results, (2) R functions for BIT implementation, and (3) source code for the R functions.

1 Additional simulation results

Table S1. Additional simulation settings for BIT designs with different response rates. Here, $p(a_0)$ and $p(a_1)$ are the true probability of a participant surviving Phase 1 of the study, and $\pi(d_{jk})$ is the overall survival probability associated with treatment combination d_{jk} for the entire trial (both phases). The family-wise type I error rate (FWER) is to be evaluated at values in the red font. GP1 is to be evaluated at values in the blue font. GP2 is to be evaluated at values in green or blue fonts.

Scenarios	$p(a_0)$	$p(a_1)$	$\boldsymbol{\pi}(d_{00})$	$\boldsymbol{\pi}(d_{01})$	$\boldsymbol{\pi}(d_{10})$	$\boldsymbol{\pi}(d_{11})$
1	0.6	0.6	0.40	0.40	0.45	0.45
2	0.6	0.7	0.40	0.40	0.45	0.60
3	0.6	0.7	0.40	0.55	0.45	0.60
4	0.6	0.7	0.40	0.55	0.55	0.60
5	0.6	0.7	0.40	0.40	0.40	0.40
6	0.7	0.6	0.40	0.50	0.55	0.55
7	0.8	0.8	0.60	0.60	0.65	0.65
8	0.8	0.9	0.60	0.60	0.65	0.80
9	0.8	0.9	0.60	0.75	0.65	0.80
10	0.8	0.9	0.60	0.75	0.75	0.80
11	0.8	0.9	0.60	0.60	0.60	0.60
12	0.9	0.8	0.60	0.70	0.75	0.75

Table S2. Simulation results: Family-wise type I error rate (FWER), generalized power 1 and 2 (GP1 and GP2) corresponding to the different simulation settings.

Scenario	FWER (%)	GP1 (%)	GP2 (%)
1	4.80		
2	0.12	80.26	80.26
3	0.02	46.24	93.30
4		38.64	92.26
5	1.42		
6		35.02	45.48
7	4.88		
8	0.42	80.74	80.74
9	0.10	50.58	90.70
10		45.10	90.24
11	1.60		
12		31.98	39.92

Table S3. Simulation results: Family-wise type I error rate (FWER), generalized power 1 and 2 (GP1 and GP2) corresponding to different survival data generating mechanisms: Exponential and Gamma distributions.

Scenario	Distribution	FWER (%)	GP1 (%)	GP2 (%)
1	Exponential	4.84		
2	Exponential	0.22	81.98	81.98
3	Exponential	0.20	48.46	93.58
4	Exponential		42.58	93.62
5	Exponential	1.94		
6	Exponential		33.54	44.60
1	Gamma	4.56		
2	Gamma	0.24	80.46	80.46
3	Gamma	0	48.14	92.40
4	Gamma		42.40	92.64
5	Gamma	1.58		
6	Gamma		34.34	43.82

Table S4. Simulation results: Family-wise type I error rate (FWER), generalized power 1 and 2 (GP1 and GP2) corresponding to different prior specifications: Beta(1,1) in the beta-binomial model and Gamma(0.1,0.1) for the hazard.

Scenario	Prior	FWER (%)	GP1 (%)	GP2 (%)
1	Beta(1,1)	5.54		
2	Beta(1,1)	0.24	81.26	81.26
3	Beta(1,1)	0.08	47.52	93.40
4	Beta(1,1)		42.56	92.50
5	Beta(1,1)	2.06		
6	Beta(1,1)		35.70	46.52
1	Gamma(0.1,0.1)	5.30		
2	Gamma(0.1,0.1)	0.24	81.66	81.66
3	Gamma(0.1,0.1)	0.12	50.02	93.40
4	Gamma(0.1,0.1)		45.16	93.08
5	Gamma(0.1,0.1)	2.48		
6	Gamma(0.1,0.1)		36.12	47.30

Table S5. Simulation results: Family-wise type I error rate (FWER), generalized power 1 and 2 (GP1 and GP2) corresponding to different numbers of intervals in the piecewise exponential model.

Scenario	Number of Intervals	FWER (%)	GP1 (%)	GP2 (%)
1	3	4.58		
2	3	0.22	80.90	80.90
3	3	0	48.86	93.58
4	3		43.46	92.04
5	3	1.32		
6	3		33.76	43.90
1	4	4.82		
2	4	0.30	81.78	81.78
3	4	0	49.40	93.84
4	4		43.76	93.42
5	4	2.02		
6	4		34.24	45.30

Table S6. Simulation results: Family-wise type I error rate (FWER), generalized power 1 and 2 (GP1 and GP2) under piecewise exponential model partitioned at different Phase 2 time points.

Scenario	Partition Point	FWER (%)	GP1 (%)	GP2 (%)
1	One-third	5.92		
2	One-third	0.30	81.26	81.26
3	One-third	0.06	48.78	94.08
4	One-third		42.74	93.60
5	One-third	2.18		
6	One-third		37.22	48.74
1	Two-thirds	6.12		
2	Two-thirds	0.28	81.38	81.38
3	Two-thirds	0.10	47.90	93.96
4	Two-thirds		42.78	93.40
5	Two-thirds	2.52		
6	Two-thirds		36.36	47.38

Table S7. Simulation results: Family-wise type I error rate (FWER), generalized power 1 and 2 (GP1 and GP2) replacing piecewise exponential model with Weibull model.

Scenario	FWER (%)	GP1 (%)	GP2 (%)
1	5.36		
2	0.28	81.88	81.88
3	0.14	48.28	93.92
4		40.52	93.34
5	1.92		
6		35.48	47.20

Table S8. Simulation results: Family-wise type I error rate (FWER), generalized power 1 and 2 (GP1 and GP2) corresponding to different numbers of interim analyses.

Scenario	Number of Interim Analyses	FWER (%)	GP1 (%)	GP2 (%)
1	1	4.66		
2	1	0.18	80.52	80.52
3	1	0.14	49.46	91.80
4	1		42.16	90.62
5	1	1.66		
6	1		39.90	49.12
1	3	4.94		
2	3	0.36	80.04	80.04
3	3	0.16	45.56	94.12
4	3		40.40	93.86
5	3	2.14		
6	3		32.12	44.16

2 Two R functions for BIT implementation

We have developed R codes to implement the BIT design through simulation. There are two main functions. The first function `BIT.Simulation` performs simulation for assessing the operating characteristics of BIT design through generated data. The arguments of `BIT.Simulation()` are:

`p1_a0`: Phase 1 survival rate of the control group

`p1_a1`: Phase 1 survival rate of the treatment group

`pi_d00`: Overall survival rate under regime d_{00}

`pi_d01`: Overall survival rate under regime d_{01}

`pi_d10`: Overall survival rate under regime d_{10}

`pi_d11`: Overall survival rate under regime d_{11}

`alpha`: Design parameter α from grid search

`delta`: Design parameter δ from grid search

`ncohort`: number of randomization cohorts

`cohortsize`: Patients per cohort (2 per arm)

`ntrial`: Number of simulated trials

`enrollrate`: Expected enrollment rate (9 patients/month)

`diff`: Minimal meaningful margin Δ

`T1`: Days by end of Phase 1

`T2`: Days by end of Phase 2

`T_pe`: First sub-interval length within phase 2

The following R codes will provide the simulation result of scenario 1 in Table 3 and 4.

```
BIT.Simulation(p1_a0 = 0.7, p1_a1 = 0.7, pi_d00 = 0.5, pi_d01 = 0.5, pi_d10
= 0.55, pi_d11 = 0.55, alpha = 0.98, delta = 0.95, ncohort = 37, cohortsize
= 8, ntrial = 5000, enrollrate = 9, diff = 0.05, T1 = 30, T2 = 360, T_pe =
165)
```

The second function `BIT` provides the optimal treatment regime selection for practical trial implementation. This function has three additional input arguments:

`npatient`: total sample size

`seed`: random number generator seed

`phase`: which phase to run (1: first interim analysis; 2: second interim analysis; 3: final analysis)

The following R codes will provide the result of first interim analysis in practice.

```
BIT(npatient=300, diff=0.05, T1=30, T2=360, T_pe=165, seed = 123, phase=1)
```

3 Source Code for the two R functions

This section include the source code for `BIT.Simulation` and `BIT` functions.

3.1 The `BIT.Simulation` function

BIT.Simulation Function

```
#####################################################################
#####          BIT.Simulation() simulation of BIT design
#####################################################################

BIT.Simulation = function(p1_a0, p1_a1, pi_d00, pi_d01, pi_d10, pi_d11,
                          alpha=0.98, delta=0.95, ncohort=37, cohortsize
                             =8, ntrial=5000,
                          enrollrate=9, diff=0.05, T1=30, T2=360, T_pe
                             =165){

##### p1_a0: stage 1 survival rate of control group
##### p1_a0: stage 1 survival rate of treatment group
##### pi_d00: overall survival rate of treatment regime d00
##### pi_d01: overall survival rate of treatment regime d01
##### pi_d10: overall survival rate of treatment regime d10
##### pi_d11: overall survival rate of treatment regime d11

## alpha: design parameter alpha from grid search results
## delta: design parameter delta from grid search results
```

```r

## Sample size = ceiling ncohort * cohortsize to nearest multiple of 10 =
    300
## ncohort: number of randomization block
## cohortsize: number of patients in each block - 2 for each treatment
## ntrial: number of simulated trial

## enrollrate: expect to enroll 9 patients per month
## diff: minimal meaningful margins Delta - >0.05 means effective

## T1: cumulative days by the end of stage 1
## T2: cumulative days by the end of stage 2
## T_pe: cumulative days in stage 2 s1 sub-interval
Tm = (T2-T1)/2 # half the duration of stage 2

p2_d00 = pi_d00 / p1_a0
p2_d01 = pi_d01 / p1_a0
p2_d10 = pi_d10 / p1_a1
p2_d11 = pi_d11 / p1_a1
p2 = c(p2_d00, p2_d01, p2_d10, p2_d11)

pm = p2 + (1-p2)*0.4 # survival rate in the first half of stage 2

wb_gamma = log(log(p2)/log(pm), (T2-T1)/Tm) # parameter gamma of Weibull
    distribution
wb_rho = -log(pm)/(Tm^wb_gamma) # parameter rho of Weibull distribution


# Simulate patients' enrollment time point from an exponential
    distribution
SimulateArrivalTimes <- function(enrollrate, npatient)
  #### enrollrate: number of patients expected to be enrolled per month
{
  enttime <- cumsum(rexp(npatient ,enrollrate)) ## unit: month
  enttime <- ceiling(enttime*30) ## convert unit to day; take the ceiling
      of the number
```

```
  return(enttime)
}


# Simulate patients at the beginning: equal randomization into 1 control
    and 3 treatment groups
SimulatePatientsI <- function(ncohortI){
  y <- c() ## whether survive in stage 1: 0 - died; 1 - survived
  z <- c() ## survival duration (unit: day) in stage 2

  trt1 <- c() ## indicating patients' group in stage 1: 0 - control; 1 -
      treatment
  trt2 <- c() ## indicating patients' group in stage 2: 0 - control; 1 -
      treatment
  trt1_values <- c(0, 1)
  trt2_values <- c(0, 1)

  for (i in 1:ncohortI){
    trt1.temp <- sample(rep(trt1_values, each = cohortsize/length(trt1_
        values))) ## assigned patients equally to stage 1 groups
    trt2.temp <- c()
    for (m in trt1_values) {
      trt2.temp[trt1.temp == m] <- sample(rep(trt2_values, each = sum(trt1
          .temp == m) / 2))
    } #assigned patients equally to stage 2 treatment under each stage 1
        group

    y.temp=rep(NA,cohortsize) ## whether survive in stage 1: yes==1, no==0
    z.temp=rep(NA,cohortsize) ## survival time in stage 2

    for (j in 1:cohortsize){
      if (trt1.temp[j] == 0){
        y.temp[j]=rbinom(1,1,p1_a0)
      } else if (trt1.temp[j] == 1){y.temp[j]=rbinom(1,1,p1_a1)}
    }

```

```
    for (j in 1:cohortsize){
      if (y.temp[j]==1) {
        if (trt1.temp[j]==0 & trt2.temp[j]==0) {
          z.temp[j]=(-log(runif(1))/wb_rho[1])^(1/wb_gamma[1])
        } else if (trt1.temp[j]==0 & trt2.temp[j]==1) {
          z.temp[j]=(-log(runif(1))/wb_rho[2])^(1/wb_gamma[2])
        } else if (trt1.temp[j]==1 & trt2.temp[j]==0) {
          z.temp[j]=(-log(runif(1))/wb_rho[3])^(1/wb_gamma[3])
        } else if (trt1.temp[j]==1 & trt2.temp[j]==1) {
          z.temp[j]=(-log(runif(1))/wb_rho[4])^(1/wb_gamma[4])}
      }
    }

    npatientI  <- ncohortI * cohortsize
    enttime=SimulateArrivalTimes(enrollrate, npatientI)
    trt1=c(trt1,trt1.temp)
    trt2=c(trt2,trt2.temp)
    y=c(y,y.temp)
    z=c(z,z.temp)

    endtime=rep(NA, npatientI)
    for(n in 1:length(enttime)){
      endtime[n]=ifelse(y[n]==0, enttime[n]+T1, pmin(enttime[n]+z[n],
          enttime[n]+T2)) #follow-up end time point
    }
  }

  return(data.frame(enttime=enttime,endtime=endtime,trt1=trt1,trt2=trt2,y=
      y,z=z))

}

# Simulate posterior probability
post.prob <- function(y,z,trt1,trt2){
  post.p1 = NULL #simulation of posterior p1
  post.p2 = NULL #simulation of posterior p2
```

```
  post.pi = NULL #simulation of posterior pi
  post.avg.p1 = rep(0, 2) #expectation of posterior p1
  post.avg.p2 = matrix(rep(0, 4), ncol = 2) #expectation of posterior p2
  post.avg.pi=matrix(rep(0, 4), ncol = 2) #expectation of posterior pi
  post.prob.pi=matrix(rep(0, 4), ncol = 2) #probability of posterior pi_jk
      > pi_00+diff

  for (j in 0:1){
    ytrt = y[trt1==j] # whether survived in stage 1 under trt1 == j, sum(
        ytrt) -> r1j

    #parameters alpha & beta for beta distribution of posterior p1
    post.alpha_b = 0.5 + sum(ytrt)
    post.beta_b = 0.5 + length(ytrt) - sum(ytrt)

    #simulation of p1
    post.p1.temp = rbeta(5000, post.alpha_b, post.beta_b)
    post.p1 = rbind(post.p1, post.p1.temp)

    #calculate expectation of beta distribution of posterior p1(aj)
    post.avg.p1[j+1] = post.alpha_b/(post.alpha_b+post.beta_b)

    for (k in 0:1){
      ztrt = z[trt1 == j & trt2 == k & y == 1] # stage 2 survival duration
          under djk, length(ztrt) -> n2jk

      upsilon_s1 = pmin(ztrt, T_pe) # survival time in s1 sub-interval of
         stage 2
      delta_s1 = ifelse(ztrt <= T_pe, 1, 0) # delta_s1 == 1 if patient
         died in s1

      ztrt_s2 = pmin(ztrt, T2-T1) # survival time in stage 2
      upsilon_s2 = ifelse((ztrt_s2-T_pe)>0, ztrt_s2-T_pe, 0) # survival
         time in s2 sub-interval of stage 2
      delta_s2 = ifelse(T_pe < ztrt & ztrt <= (T2-T1), 1, 0) # delta_s2 ==
          1 if patient died in s2
```

```
143
144       post.alpha_g_s1 = 0.01 + sum(delta_s1) # parameter alpha of gamma
              distribution of posterior lambda_{jk1}
145       post.beta_g_s1 = 0.01 + sum(upsilon_s1) # parameter beta of gamma
              distribution of posterior lambda_{jk1}
146       post.alpha_g_s2 = 0.01 + sum(delta_s2) # parameter alpha of gamma
              distribution of posterior lambda_{jk2}
147       post.beta_g_s2 = 0.01 + sum(upsilon_s2) # parameter beta of gamma
              distribution of posterior lambda_{jk2}
148
149       #simulation of pi
150       post.lambda_exp_s1 = rgamma(5000,post.alpha_g_s1,rate=post.beta_g_s1
              )
151       post.lambda_exp_s2 = rgamma(5000,post.alpha_g_s2,rate=post.beta_g_s2
              )
152       post.p2.temp = exp(-post.lambda_exp_s1*T_pe -post.lambda_exp_s2*(T2-
              T_pe-T1))
153       post.pi.temp = post.p1.temp * post.p2.temp
154
155       post.p2 = rbind(post.p2, post.p2.temp)
156       post.pi = rbind(post.pi, post.pi.temp)
157
158       #mean of p2 and pi
159       post.avg.p2_s1 = (post.beta_g_s1/(post.beta_g_s1+T_pe))^post.alpha_g
              _s1
160       post.avg.p2_s2 = (post.beta_g_s2/(post.beta_g_s2+T2-T_pe-T1))^post.
              alpha_g_s2
161       post.avg.p2[j+1,k+1]=post.avg.p2_s1 * post.avg.p2_s2
162       post.avg.pi[j+1,k+1]=post.avg.p1[j+1] * post.avg.p2[j+1,k+1]
163     }
164   }
165
166   # PP(d_{jk}) -- probability of posterior pi_{djk} > pi_{00} + diff
167   a=1
168   for (j in 0:1){
169     for (k in 0:1){
```

```
      if(j==0 & k==0) {post.prob.pi[j+1,k+1]=0
      } else {post.prob.pi[j+1,k+1]=mean(as.integer(post.pi[a,]>post.pi
          [1,]+diff))}
      a=a+1
    }
  }

  return(list(post.avg.p1=post.avg.p1,post.avg.p2=post.avg.p2,post.avg.pi=
      post.avg.pi,post.prob.pi=post.prob.pi))
}


# In interim analysis, define early stopping boundary and construct
    candidate integrated set - zeta, select optimal therapy
InterimAnalysis <- function(post.prob.pi, intrm.npatient,npatient){

  zeta.index=matrix(rep(0, 4), ncol = 2)
  dr.opt.index=matrix(rep(0, 4), ncol = 2) # index of optimal therapy
  futility.stop=NA
  superiority.stop=NA

  futility.cutoff = delta*(intrm.npatient/npatient)^alpha # lower boundary
      for stopping

  q = (1+delta)/2 #upper quantile of Z in superiority cutoff
  superiority.cutoff = 2*pnorm(qnorm(q, mean = 0, sd = 1)/sqrt(intrm.
      npatient/npatient), mean = 0, sd = 1)-1 # upper boundary for stopping

  for(j in 0:1){
    for(k in 0:1){
      if(j==0 & k==0) {zeta.index[j+1,k+1]=0
      } else {zeta.index[j+1,k+1] = ifelse(post.prob.pi[j+1,k+1]>futility.
          cutoff, 1, 0)}
    }
  }

```

```
  futility.stop=ifelse(sum(zeta.index)==0, 1, 0) #early stop for futility:
      yes==1

  if(futility.stop == 0){
    dr.opt.position = which(zeta.index == 1 & post.prob.pi==max(post.prob.
       pi), arr.ind = TRUE) # get the position of optimal therapy in the 2
        by 2 matrix
    dr.opt.index[dr.opt.position[1], dr.opt.position[2]] = 1
    superiority.stop=ifelse(post.prob.pi[dr.opt.position[1], dr.opt.
       position[2]]>superiority.cutoff, 1, 0) #early stop for superiority:
        yes==1
  } else {dr.opt.index[1,1]=1}

  return(list(futility.stop=futility.stop, superiority.stop=superiority.
     stop, zeta.index=zeta.index, dr.opt.index=dr.opt.index))
}


# Simulate patients: equally assigned to candidate set treatments
SimulatePatientsII <- function(ncohortII, zeta.index){
  zeta.index[1,1]=1 #assign patients to candidate treatment group with
     index==1
  trt.position <- which(zeta.index == 1, arr.ind = TRUE) #identify
     position with trt index==1
  cohortsizeII = sum(zeta.index)*2

  y <- c() # whether survive in stage 1, survive == 1
  z <- c() # survival duration in stage 2
  trt1 <- c()
  trt2 <- c()
  trt.row = c()
  trt.col = c()
  assigned.indices=c()
  assigned.trt=c()

  npatientII  <- ncohortII * cohortsizeII
```

```
  enttime=SimulateArrivalTimes(enrollrate, npatientII)
  endtime=rep(NA, npatientII)

  for (i in 1:ncohortII){

    trt1.temp=rep(NA,cohortsizeII)
    trt2.temp=rep(NA,cohortsizeII)
    y.temp=rep(NA,cohortsizeII) ## whether survive in stage 1: yes==1, no
        ==0
    z.temp=rep(NA,cohortsizeII) ## survival time in stage 2

    assigned.indices.temp <- rep(1:nrow(trt.position), each = 2) # assign
        2 patients to each treatment in a block
    assigned.indices.temp <- sample(assigned.indices.temp) ## shuffle the
        assignment order
    assigned.trt.temp <- trt.position[assigned.indices.temp, ]

    trt.row.temp=assigned.trt.temp[, 1]
    trt.col.temp=assigned.trt.temp[, 2]

    trt1.temp=ifelse(trt.row.temp==1, 0, 1) #first trt row: pi_00 or pi_
        01, trt in stage 1 is control
    trt2.temp=ifelse(trt.col.temp==1, 0, 1) #first trt col: pi_00, pi_10,
        trt in stage 2 is control

    for (j in 1:cohortsizeII){
      if (trt1.temp[j] == 0){
        y.temp[j]=rbinom(1,1,p1_a0)
      } else if (trt1.temp[j] == 1){y.temp[j]=rbinom(1,1,p1_a1)}
    }

    for (j in 1:cohortsizeII){
      if (y.temp[j]==1) {
        if (trt1.temp[j]==0 & trt2.temp[j]==0) {
          z.temp[j]=(-log(runif(1))/wb_rho[1])^(1/wb_gamma[1])
        } else if (trt1.temp[j]==0 & trt2.temp[j]==1) {
```

```
259             z.temp[j]=(-log(runif(1))/wb_rho[2])^(1/wb_gamma[2])
260           } else if (trt1.temp[j]==1 & trt2.temp[j]==0) {
261             z.temp[j]=(-log(runif(1))/wb_rho[3])^(1/wb_gamma[3])
262           } else if (trt1.temp[j]==1 & trt2.temp[j]==1) {
263             z.temp[j]=(-log(runif(1))/wb_rho[4])^(1/wb_gamma[4])}
264         }
265       }
266
267       assigned.trt=rbind(assigned.trt,assigned.trt.temp)
268       assigned.indices=c(assigned.indices,assigned.indices.temp)
269       trt.row=c(trt.row,trt.row.temp)
270       trt.col=c(trt.col,trt.col.temp)
271       trt1=c(trt1,trt1.temp)
272       trt2=c(trt2,trt2.temp)
273       y=c(y,y.temp)
274       z=c(z,z.temp)
275
276     }
277     for(n in 1:npatientII){
278       endtime[n]=ifelse(y[n]==0, enttime[n]+T1, pmin(enttime[n]+z[n],
            enttime[n]+T2)) #follow-up end time point
279     }
280     return(data.frame(assigned.indices=assigned.indices,enttime=enttime,
          endtime=endtime,assigned.trt=assigned.trt,trt1=trt1,trt2=trt2,y=y,z=z
          ))
281   }
282
283   # Repeat trials for ntrial times and select optimal therapy
284   TreatmentDecision <- function(alpha, delta, ncohort){
285
286     opt.final <- c()
287     early.stop <- c()
288     early.stop.ft <- c() # index of early stop for futility
289     early.stop.sup <- c() # index of early stop for superority
290     sample.size <- c()
291     npatient  <- ceiling(ncohort * cohortsize/ 10) * 10
```

```
292   ncohortI = ceiling(ncohort*0.6)
293   ncohortII = ceiling(ncohort*0.8)
294
295   for(i in 1:ntrial){
296
297     opt=rep(0,4)
298     early.stop.temp=0
299     early.stop.ft.temp=0
300     early.stop.sup.temp=0
301     sample.size.temp=NA
302
303     df1 = SimulatePatientsI(ncohortI)
304     intrm.npatientI = floor(0.3 * npatient)
305     dfI = df1[order(df1$endtime), ][1:intrm.npatientI, ] #get first 30%
          patients for interim analysis
306     pp1=post.prob(dfI$y,dfI$z,dfI$trt1,dfI$trt2)
307     r1=InterimAnalysis(pp1$post.prob.pi, intrm.npatientI,npatient)
308
309     if(r1$futility.stop==1){
310       opt[1] = opt[1]+1
311       early.stop.temp = early.stop.temp+1
312       early.stop.ft.temp = early.stop.ft.temp+1
313       sample.size.temp = intrm.npatientI
314     } else if(r1$superiority.stop == 1){
315       early.stop.temp = early.stop.temp+1
316       early.stop.sup.temp = early.stop.sup.temp+1
317       sample.size.temp = intrm.npatientI
318
319       opt.position <- which(r1$dr.opt.index == 1, arr.ind = TRUE)
320       if(opt.position[,1]==1 & opt.position[,2]==2){
321         opt[2] = opt[2]+1 ##pi_01
322       }else if(opt.position[,1]==2 & opt.position[,2]==1){
323         opt[3] = opt[3]+1 ##pi_10
324       }else if(opt.position[,1]==2 & opt.position[,2]==2){opt[4] = opt
            [4]+1} ##pi_11
325
```

```
326     } else{
327
328       df2=SimulatePatientsII(ncohortII,r1$zeta.index)
329       intrm.npatientII = floor((0.7 - 0.3) * npatient)
330       dfII = df2[order(df2$endtime), ][1:intrm.npatientII, -which(names(
              df2) %in% c("assigned.indices","assigned.trt.row","assigned.trt.
              col"))] #get 30-70% patients for interim analysis
331       dfII =rbind(dfI, dfII)
332
333       pp2=post.prob(dfII$y,dfII$z,dfII$trt1,dfII$trt2)
334       r2=InterimAnalysis(pp2$post.prob.pi, intrm.npatientI+intrm.
              npatientII, npatient)
335
336       if(r2$futility.stop==1){
337         opt[1] = opt[1]+1
338         early.stop.temp = early.stop.temp+1
339         early.stop.ft.temp = early.stop.ft.temp +1
340         sample.size.temp = intrm.npatientI+intrm.npatientII
341       } else if(r2$superiority.stop == 1){
342         early.stop.temp = early.stop.temp+1
343         early.stop.sup.temp = early.stop.sup.temp +1
344         sample.size.temp = intrm.npatientI+intrm.npatientII
345
346         opt.position <- which(r2$dr.opt.index == 1, arr.ind = TRUE)
347         if(opt.position[,1]==1 & opt.position[,2]==2){
348           opt[2] = opt[2]+1 ##pi_01
349         }else if(opt.position[,1]==2 & opt.position[,2]==1){
350           opt[3] = opt[3]+1 ##pi_10
351         }else if(opt.position[,1]==2 & opt.position[,2]==2){opt[4] = opt
              [4]+1} ##pi_11
352
353       }else{
354
355         df3=SimulatePatientsII(ncohort,r2$zeta.index)
356         intrm.npatientIII = npatient - (intrm.npatientI + intrm.npatientII
              )
```

```
        dfIII = df3[order(df3$endtime), ][1:intrm.npatientIII, -which(
            names(df3) %in% c("assigned.indices","assigned.trt.row","
            assigned.trt.col"))] #get 70-100% patients for interim analysis
        dfIII = rbind(dfII, dfIII)

        pp3=post.prob(dfIII$y,dfIII$z,dfIII$trt1,dfIII$trt2)
        r3=InterimAnalysis(pp3$post.prob.pi, npatient, npatient)
        sample.size.temp = npatient

        opt.position <- which(r3$dr.opt.index == 1, arr.ind = TRUE)
        if(opt.position[,1]==1 & opt.position[,2]==2){
          opt[2] = opt[2]+1 ##pi_01
        }else if(opt.position[,1]==2 & opt.position[,2]==1){
          opt[3] = opt[3]+1 ##pi_10
        }else if(opt.position[,1]==2 & opt.position[,2]==2){opt[4] = opt
            [4]+1}##pi_11
      }
    }
    opt.final=rbind(opt.final, opt)
    early.stop = rbind(early.stop, early.stop.temp)
    early.stop.ft = rbind(early.stop.ft, early.stop.ft.temp)
    early.stop.sup = rbind(early.stop.sup, early.stop.sup.temp)
    sample.size = rbind(sample.size, sample.size.temp)
  }
  return(data.frame(opt.final, early.stop, sample.size, early.stop.ft,
      early.stop.sup))
}

# Simulate results for ntrial=5000 trials
set.seed(123)
opt.rslt <- TreatmentDecision(alpha, delta, ncohort)
opt.rslt <- data.frame(opt.rslt)

# --------decision block for type I error, GP1 & GP2--------------
rslt <- matrix(nrow = 1, ncol = 7)
```

```r
colnames(rslt) <- c("type I error", "GP1", "GP2", "avg sample size","early stopping prob",
                    "f stop prob", "s stop prob")

# ---map each treatment regime pi to its  r e p number
pi_vals   <- c(pi_d01, pi_d10, pi_d11)
rep_cols  <- 2:4

# treatment regimes count as type I error
typeI_pi <- pi_vals <= (pi_d00 + diff)
typeI_col <- rep_cols[typeI_pi]

if (sum(typeI_col) == 0) {
  rslt[1, "type I error"] <- NA
} else {
  rslt[1, "type I error"] <- sum(opt.rslt[, typeI_col]) / ntrial
}

# treatment regimes count as GP1 and GP2
eff_col <- rep_cols[!typeI_pi]
eff_val <- pi_vals[!typeI_pi]

if (sum(eff_col) == 0) {
  rslt[1, "GP1"] <- NA
  rslt[1, "GP2"] <- NA
} else{
  best_idx <- which(eff_val == max(eff_val))
  best_col <- eff_col[best_idx]

  rslt[1, "GP1"] <- sum(opt.rslt[, best_col]) / ntrial
  rslt[1, "GP2"] <- sum(opt.rslt[, eff_col]) / ntrial
 }

rslt[1,"avg sample size"] <- sum(opt.rslt[, 6]) / ntrial
rslt[1,"early stopping prob"] <- sum(opt.rslt[, 5]) / ntrial
```

```
rslt[1,"f stop prob"] <- sum(opt.rslt[, 7])/ntrial #prob of stopping for
    futility
rslt[1,"s stop prob"] <- sum(opt.rslt[, 8])/ntrial #prob of stopping for
    superiority

return(data.frame(rslt))
}

##### Example: Practical trial implementation as shown in Table 4

## Scenario 1
sce1 = BIT.Simulation(p1_a0 = 0.7, p1_a1 = 0.7, pi_d00 = 0.5, pi_d01 =
    0.5, pi_d10 = 0.55, pi_d11 = 0.55)

## Scenario 2
sce2 = BIT.Simulation(p1_a0 = 0.7, p1_a1 = 0.8, pi_d00 = 0.5, pi_d01 =
    0.5, pi_d10 = 0.55, pi_d11 = 0.70)

## Scenario 3
sce3 = BIT.Simulation(p1_a0 = 0.7, p1_a1 = 0.8, pi_d00 = 0.5, pi_d01 =
    0.65, pi_d10 = 0.55, pi_d11 = 0.7)

## Scenario 4
sce4 = BIT.Simulation(p1_a0 = 0.7, p1_a1 = 0.8, pi_d00 = 0.5, pi_d01 =
    0.65, pi_d10 = 0.65, pi_d11 = 0.7)

## Scenario 5
sce5 = BIT.Simulation(p1_a0 = 0.7, p1_a1 = 0.8, pi_d00 = 0.5, pi_d01 =
    0.5, pi_d10 = 0.5, pi_d11 = 0.5)

## Scenario 6
sce6 = BIT.Simulation(p1_a0 = 0.8, p1_a1 = 0.7, pi_d00 = 0.5, pi_d01 =
    0.6, pi_d10 = 0.65, pi_d11 = 0.65)
```

3.2 The BIT function

BIT Function

```
##### input table should have variables: enttime, endtime,trt1,trt2,y,z
## enttime: patient enrollment time
## endtime: time of patient death or censor
## trt1: indicating patients' group in stage 1: 0 - control; 1 - treatment
## trt2: indicating patients' group in stage 2: 0 - control; 1 - treatment
## y: whether survive in stage 1: 0 - died; 1 - survived
## z: survival duration (unit: day) in stage 2

##### input tables: df1, df2, df3
## df1: patients recruited before first interim analysis (around 30% of
    sample size)
## df2: patients' recruited between first and second interim analysis (
    around 40% of sample size)
## df3: patients recruited after second interim analysis (around 30% of
    sample size)

#-----------------------------------------------------------------------
# BIT implement function start
#-----------------------------------------------------------------------
BIT = function(npatient, diff, T1, T2, T_pe, seed, phase, alpha=0.98,
    delta=0.95,ntrial=5000){

  ##### npatient: total sample size
  ##### diff:  minimal meaningful margins delta, in paper we defined delta
      >0.05 -> effective
  ##### T1: cumulative days by the end of stage 1, in paper we used 30
  ##### T2: cumulative days by the end of stage 2, in paper we used 360
  ##### T_pe: cumulative days in stage 2 s1 subinterval, in paper we used
      165
  ##### seed: random number generator seed
  ##### phase: which phase to run (1: only df1, 2: df1+df2, 3: df1+df2+df3
      )

  ## alpha: design parameter alpha from grid search results
  ## delta: design parameter delta from grid search results
```

```
29
30
31 #-------------------------------------------------------------------------
32 #  Function to simulate posterior probability according to input data
33 #-------------------------------------------------------------------------
34   post.prob <- function(y,z,trt1,trt2){
35     post.p1 = NULL #simulation of posterior p1
36     post.p2 = NULL #simulation of posterior p2
37     post.pi = NULL #simulation of posterior pi
38     post.avg.p1 = rep(0, 2) #expectation of posterior p1
39     post.avg.p2 = matrix(rep(0, 4), ncol = 2) #expectation of posterior p2
40     post.avg.pi=matrix(rep(0, 4), ncol = 2) #expectation of posterior pi
41     post.prob.pi=matrix(rep(0, 4), ncol = 2) #probability of posterior pi_
         jk > pi_00+diff
42
43     for (j in 0:1){
44       ytrt = y[trt1==j] # whether survived in stage 1 under trt1 == j, sum
           (ytrt) -> r1j
45
46       #parameters alpha & beta for beta distribution of posterior p1
47       post.alpha_b = 0.5 + sum(ytrt)
48       post.beta_b = 0.5 + length(ytrt) - sum(ytrt)
49
50       #simulation of p1
51       post.p1.temp = rbeta(5000, post.alpha_b, post.beta_b)
52       post.p1 = rbind(post.p1, post.p1.temp)
53
54       #calculate expectation of beta distribution of posterior p1(aj)
55       post.avg.p1[j+1] = post.alpha_b/(post.alpha_b+post.beta_b)
56
57       for (k in 0:1){
58         ztrt = z[trt1 == j & trt2 == k & y == 1] # stage 2 survival
             duration under djk, length(ztrt) -> n2jk
59
60         upsilon_s1 = pmin(ztrt, T_pe) # survival time in s1 sub-interval
             of stage 2
```

```
61          delta_s1 = ifelse(ztrt <= T_pe, 1, 0) # delta_s1 == 1 if patient
                died in s1
62
63          ztrt_s2 = pmin(ztrt, T2-T1) # survival time in stage 2
64          upsilon_s2 = ifelse((ztrt_s2-T_pe)>0, ztrt_s2-T_pe, 0) # survival
                time in s2 sub-interval of stage 2
65          delta_s2 = ifelse(T_pe < ztrt & ztrt <= (T2-T1), 1, 0) # delta_s2
                == 1 if patient died in s2
66
67          post.alpha_g_s1 = 0.01 + sum(delta_s1) # parameter alpha of gamma
                distribution of posterior lambda_{jk1}
68          post.beta_g_s1 = 0.01 + sum(upsilon_s1) # parameter beta of gamma
                distribution of posterior lambda_{jk1}
69          post.alpha_g_s2 = 0.01 + sum(delta_s2) # parameter alpha of gamma
                distribution of posterior lambda_{jk2}
70          post.beta_g_s2 = 0.01 + sum(upsilon_s2) # parameter beta of gamma
                distribution of posterior lambda_{jk2}
71
72          #simulation of pi
73          post.lambda_exp_s1 = rgamma(5000,post.alpha_g_s1,rate=post.beta_g_
                s1)
74          post.lambda_exp_s2 = rgamma(5000,post.alpha_g_s2,rate=post.beta_g_
                s2)
75          post.p2.temp = exp(-post.lambda_exp_s1*T_pe -post.lambda_exp_s2*(
                T2-T_pe-T1))
76          post.pi.temp = post.p1.temp * post.p2.temp
77
78          post.p2 = rbind(post.p2, post.p2.temp)
79          post.pi = rbind(post.pi, post.pi.temp)
80
81          #mean of p2 and pi
82          post.avg.p2_s1 = (post.beta_g_s1/(post.beta_g_s1+T_pe))^post.alpha
                _g_s1
83          post.avg.p2_s2 = (post.beta_g_s2/(post.beta_g_s2+T2-T_pe-T1))^post
                .alpha_g_s2
84          post.avg.p2[j+1,k+1]=post.avg.p2_s1 * post.avg.p2_s2
```

```
85          post.avg.pi[j+1,k+1]=post.avg.p1[j+1] * post.avg.p2[j+1,k+1]
86        }
87      }
88
89      # PP(d_{jk}) -- probability of posterior pi_{djk} > pi_{00} + diff
90      a=1
91      for (j in 0:1){
92        for (k in 0:1){
93          if(j==0 & k==0) {post.prob.pi[j+1,k+1]=0
94          } else {post.prob.pi[j+1,k+1]=mean(as.integer(post.pi[a,]>post.pi
                [1,]+diff))}
95          a=a+1
96        }
97      }
98
99      return(list(post.avg.p1=post.avg.p1,post.avg.p2=post.avg.p2,post.avg.
            pi=post.avg.pi,post.prob.pi=post.prob.pi))
100   }
101
102
103 #-------------------------------------------------------------------------
104 # Interim analysis function to decide early stopping and optimal therapy
105 #-------------------------------------------------------------------------
106   InterimAnalysis <- function(post.prob.pi, intrm.npatient,npatient){
107
108     zeta.index=matrix(rep(0, 4), ncol = 2)
109     dr.opt.index=matrix(rep(0, 4), ncol = 2) # index of optimal therapy
110     futility.stop=NA
111     superiority.stop=NA
112
113     futility.cutoff = delta*(intrm.npatient/npatient)^alpha # lower
            boundary for stopping
114
115     q = (1+delta)/2 #upper quantile of Z in superiority cutoff
116     superiority.cutoff = 2*pnorm(qnorm(q, mean = 0, sd = 1)/sqrt(intrm.
            npatient/npatient), mean = 0, sd = 1)-1 # upper boundary for
```

```
        stopping
117
118     for(j in 0:1){
119       for(k in 0:1){
120         if(j==0 & k==0) {zeta.index[j+1,k+1]=0
121         } else {zeta.index[j+1,k+1] = ifelse(post.prob.pi[j+1,k+1]>
                futility.cutoff, 1, 0)}
122       }
123     }
124
125     futility.stop=ifelse(sum(zeta.index)==0, 1, 0) #early stop for
          futility: yes==1
126
127     if(futility.stop == 0){
128       dr.opt.position = which(zeta.index == 1 & post.prob.pi==max(post.
            prob.pi), arr.ind = TRUE) # get the position of optimal therapy
            in the 2 by 2 matrix
129       dr.opt.index[dr.opt.position[1], dr.opt.position[2]] = 1
130       superiority.stop=ifelse(post.prob.pi[dr.opt.position[1], dr.opt.
            position[2]]>superiority.cutoff, 1, 0) #early stop for
            superiority: yes==1
131     } else {dr.opt.index[1,1]=1}
132
133     return(list(futility.stop=futility.stop, superiority.stop=superiority.
          stop, zeta.index=zeta.index, dr.opt.index=dr.opt.index))
134   }
135
136 #-------------------------------------------------------------------------
137 # Reporting function: prints out optimal therapy and stopping messages
138 #-------------------------------------------------------------------------
139   report_rslt <- function(opt, early.stop, sample.size, early.stop.ft,
        early.stop.sup) {
140
141     if (early.stop == 0) {
142       cat("No early stopping\n")
143     } else {
```

```
144       if (early.stop.ft == 1) {
145         cat(paste0("Early stopping for futility, actual used sample size
              is ", sample.size, "\n"))
146       }
147       if (early.stop.sup == 1) {
148         cat(paste0("Early stopping for superiority, actual used sample
              size is ", sample.size, "\n"))
149       }
150     }
151
152     if (early.stop.ft == 1 || early.stop.sup == 1) {
153       if (opt["opt_01"] == 0 && opt["opt_10"] == 0 && opt["opt_11"] == 0)
            {
154         cat("No optimal therapy\n")
155       } else {
156         if (opt["opt_01"] == 1) cat("The optimal therapy is d01\n")
157         if (opt["opt_10"] == 1) cat("The optimal therapy is d10\n")
158         if (opt["opt_11"] == 1) cat("The optimal therapy is d11\n")
159       }
160     }
161   }
162
163   report_rslt2 <- function(opt) {
164     if (opt["opt_01"] == 0 && opt["opt_10"] == 0 && opt["opt_11"] == 0) {
165       cat("No optimal therapy\n")
166     } else {
167       if (opt["opt_01"] == 1) cat("The optimal therapy is d01\n")
168       if (opt["opt_10"] == 1) cat("The optimal therapy is d10\n")
169       if (opt["opt_11"] == 1) cat("The optimal therapy is d11\n")
170     }
171   }
172
173 #-------------------------------------------------------------------------
174 #         Treatment Decision function
175 #-------------------------------------------------------------------------
176   TreatmentDecision <- function(alpha, delta, phase){
```

```
177
178
179     opt <- setNames(rep(0,4), c("opt_00", "opt_01", "opt_10", "opt_11")) #
            index of optimal therapy
180     early.stop=0      # index of early stop
181     early.stop.ft=0   # index of early stop for futility
182     early.stop.sup=0 # index of early stop for superority
183     sample.size=NA    # actual sample size used
184
185     if(phase == 1) {
186     dfI = data.frame(df1)
187     intrm.npatientI = nrow(dfI)
188     pp1=post.prob(dfI$y, dfI$z, dfI$trt1, dfI$trt2)
189     r1=InterimAnalysis(pp1$post.prob.pi, intrm.npatientI, npatient)
190     sample.size <- intrm.npatientI
191
192     if(r1$futility.stop == 1) {
193       early.stop <- 1; early.stop.ft <- 1
194       opt.position <- which(r1$dr.opt.index == 1, arr.ind = TRUE)
195
196         if(opt.position[, 1] == 1 & opt.position[, 2] == 2) {
197           opt[2] <- opt[2] + 1
198         } else if(opt.position[, 1] == 2 & opt.position[, 2] == 1) {
199           opt[3] <- opt[3] + 1
200         } else if(opt.position[, 1] == 2 & opt.position[, 2] == 2) {
201           opt[4] <- opt[4] + 1
202         }
203
204       report_rslt(opt, early.stop, sample.size, early.stop.ft, early.stop.
              sup)
205     } else if(r1$superiority.stop == 1){
206       early.stop <- 1; early.stop.sup <- 1
207       opt.position <- which(r1$dr.opt.index == 1, arr.ind = TRUE)
208
209       if(opt.position[,1]==1 & opt.position[,2]==2){
210         opt[2] = opt[2]+1 ##pi_01
```

```
211       }else if(opt.position[,1]==2 & opt.position[,2]==1){
212         opt[3] = opt[3]+1 ##pi_10
213       }else if(opt.position[,1]==2 & opt.position[,2]==2){opt[4] = opt
              [4]+1} ##pi_11
214       report_rslt(opt, early.stop, sample.size, early.stop.ft, early.stop.
              sup)
215     } else{
216
217       zeta <- r1$zeta.index
218
219       pos <- which(zeta == 1, arr.ind = TRUE)
220       c_set <- apply(pos, 1, function(idx) {
221         paste0("d", idx["row"] - 1, idx["col"] - 1)
222       })
223
224       cat("No stopping\n")
225       cat("Candidate set: ", paste(c_set, collapse = ", "), "\n", sep = ""
              )
226       cat("Current sample size is", sample.size, "\n")
227     }
228
229     } else if(phase == 2) {
230       # Use df1 and df2
231       dfI <- data.frame(df1)
232       intrm.npatientI <- nrow(dfI)
233       dfII <- data.frame(df2)
234       intrm.npatientII <- nrow(dfII)
235
236       dfII =rbind(dfI, dfII)
237
238       pp2=post.prob(dfII$y,dfII$z,dfII$trt1,dfII$trt2)
239       r2=InterimAnalysis(pp2$post.prob.pi, intrm.npatientI+intrm.
              npatientII, npatient)
240       sample.size <- intrm.npatientI + intrm.npatientII
241
242       if(r2$futility.stop==1){
```

```
243         early.stop <- 1; early.stop.ft <- 1
244         opt.position <- which(r2$dr.opt.index == 1, arr.ind = TRUE)
245
246         if(opt.position[, 1] == 1 & opt.position[, 2] == 2) {
247           opt[2] <- opt[2] + 1
248         } else if(opt.position[, 1] == 2 & opt.position[, 2] == 1) {
249           opt[3] <- opt[3] + 1
250         } else if(opt.position[, 1] == 2 & opt.position[, 2] == 2) {
251           opt[4] <- opt[4] + 1
252         }
253         report_rslt(opt, early.stop, sample.size, early.stop.ft, early.
              stop.sup)
254
255       } else if(r2$superiority.stop == 1){
256         early.stop <- 1; early.stop.sup <- 1
257         opt.position <- which(r2$dr.opt.index == 1, arr.ind = TRUE)
258
259         if(opt.position[,1]==1 & opt.position[,2]==2){
260           opt[2] = opt[2]+1 ##pi_01
261         }else if(opt.position[,1]==2 & opt.position[,2]==1){
262           opt[3] = opt[3]+1 ##pi_10
263         }else if(opt.position[,1]==2 & opt.position[,2]==2){opt[4] = opt
              [4]+1} ##pi_11
264
265         report_rslt(opt, early.stop, sample.size, early.stop.ft, early.
              stop.sup)
266
267       }else{
268
269         zeta <- r2$zeta.index
270         pos <- which(zeta == 1, arr.ind = TRUE)
271         c_set <- apply(pos, 1, function(idx) {
272           paste0("d", idx["row"] - 1, idx["col"] - 1)
273         })
274
275         cat("No stopping\n")
```

```
276         cat("Candidate set: ", paste(c_set, collapse = ", "), "\n", sep =
                ")
277         cat("Current sample size is", sample.size, "\n")
278       }
279
280     } else if(phase == 3) {
281       # Use df1, df2, and df3
282         dfI <- data.frame(df1)
283         intrm.npatientI <- nrow(dfI)
284         dfII <- data.frame(df2)
285         intrm.npatientII <- nrow(dfII)
286         dfIII <- data.frame(df3)
287         intrm.npatientIII <- nrow(dfIII)
288
289         dfIII = rbind(dfI, dfII, dfIII)
290
291         pp3=post.prob(dfIII$y,dfIII$z,dfIII$trt1,dfIII$trt2)
292         r3=InterimAnalysis(pp3$post.prob.pi, npatient, npatient)
293         sample.size = npatient
294
295         opt.position <- which(r3$dr.opt.index == 1, arr.ind = TRUE)
296         if(opt.position[,1]==1 & opt.position[,2]==2){
297           opt[2] = opt[2]+1 ##pi_01
298         }else if(opt.position[,1]==2 & opt.position[,2]==1){
299           opt[3] = opt[3]+1 ##pi_10
300         }else if(opt.position[,1]==2 & opt.position[,2]==2){opt[4] = opt
                [4]+1}##pi_11
301
302         report_rslt2(opt)
303       }
304   }
305
306 #-------------------------------------------------------------------------
307 #   Run analysis according to phase
308 #-------------------------------------------------------------------------
309   set.seed(seed)
```

```
  opt.rslt <- TreatmentDecision(alpha, delta, phase)
}

##### Example: select the optimal therapy using df1, df2, df3 from
    scenario 4
df1 = read.csv("path/test_df1.csv")
df2 = read.csv("path/test_df2.csv")
df3 = read.csv("path/test_df3.csv")

##### phase: which phase to run (1: only df1, 2: df1+df2, 3: df1+df2+df3)
BIT(npatient=300, diff=0.05, T1=30, T2=360, T_pe=165, seed = 12, phase=2)
```
